# Supplementary material for: The Brownian dynamics simulator PyRID for reacting and interacting particles written in Python
Source: Cell Rep Methods. 2025 Sep 18;5(10):101182. doi: 10.1016/j.crmeth.2025.101182 (PMC12570352; doi:10.1016/j.crmeth.2025.101182)
Supplement: Document S1. Figures S1–S4 [file mmc1.pdf]

Cell Reports Methods, Volume *5*

Supplemental information

The Brownian dynamics simulator PyRID for reacting and interacting particles written in Python

Moritz Becker, Nahid Safari, and Christian Tetzlaff

Supplementary Document S1

The Brownian dynamics simulator PyRID for reacting and interacting particles written in Python

Moritz Becker, Nahid Safari, and Christian Tetzlaff

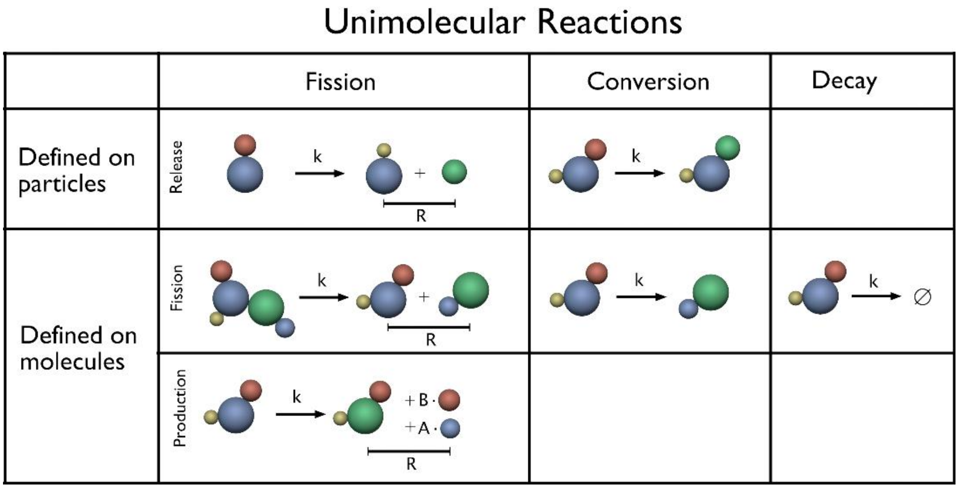

Figure S1. Schematic of unimolecular reactions. Related to Figure 6.
Unimolecular reactions can be either defined on a particle or on a molecule type. Release reactions are a sub-category of the fission reaction. Here, a molecule (rigid bead model) is released from another molecular particle/bead. Release reactions can be used, for example, to model the release of a ligand from a specific molecule’s binding site. The production reaction enables a fission reaction with more than two products. It can be used, for example, to model the influx of ions into a compartment via an ion channel.

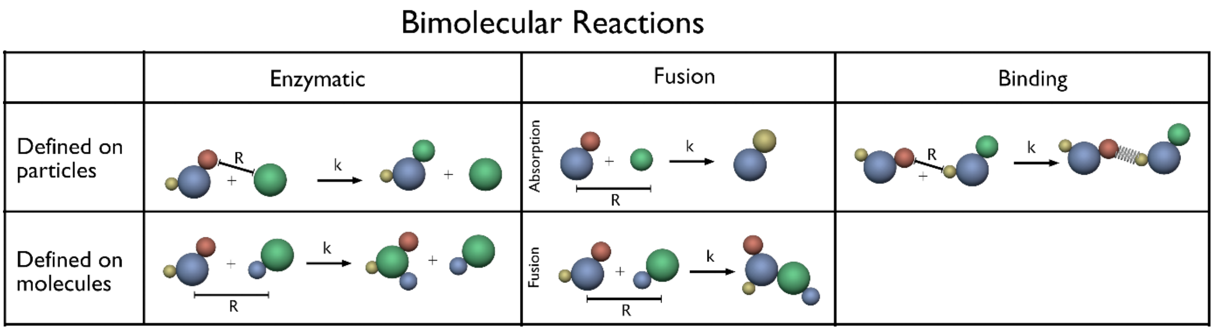

Figure S2. Schematic of bimolecular reactions. Related to Figure 6.
Bimolecular reactions can be either defined on a particle or on a molecule type. The absorption reaction is a sub-category of the fusion reaction. It has been introduced as the inverse to the release reaction and can be used to model, e.g., ligand binding. Here, a molecule is absorbed by the bead/particle of another molecule and thus removed from the simulation. Binding reactions introduce an energy potential between two particles/beads. Short-range interactions can be used, for example, to model patchy particles.

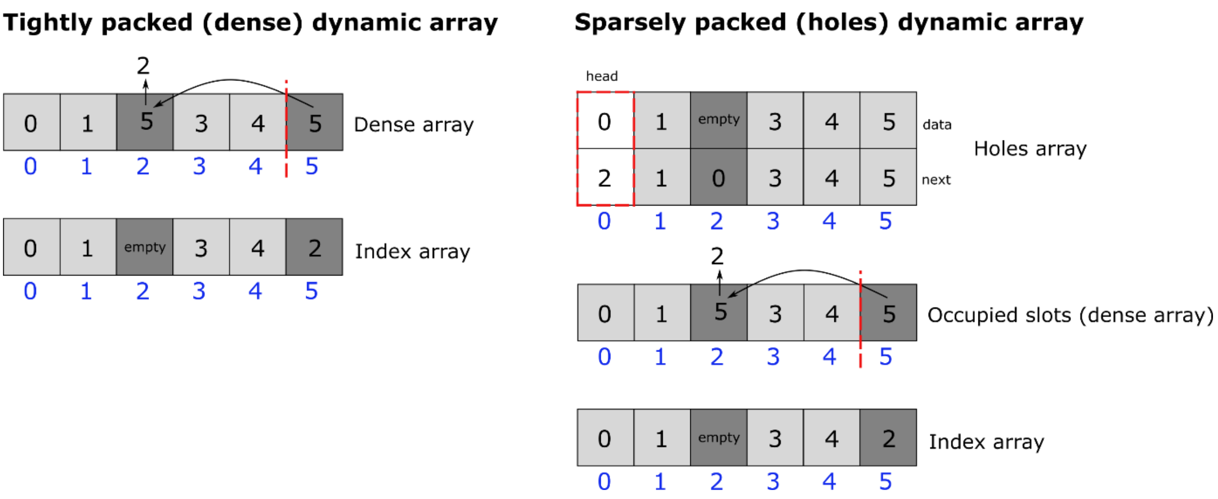

Figure S3. Dynamic arrays. Related to STAR Methods.
(left) Tightly packed dynamic array. (right) Sparsely packed dynamic arrays.

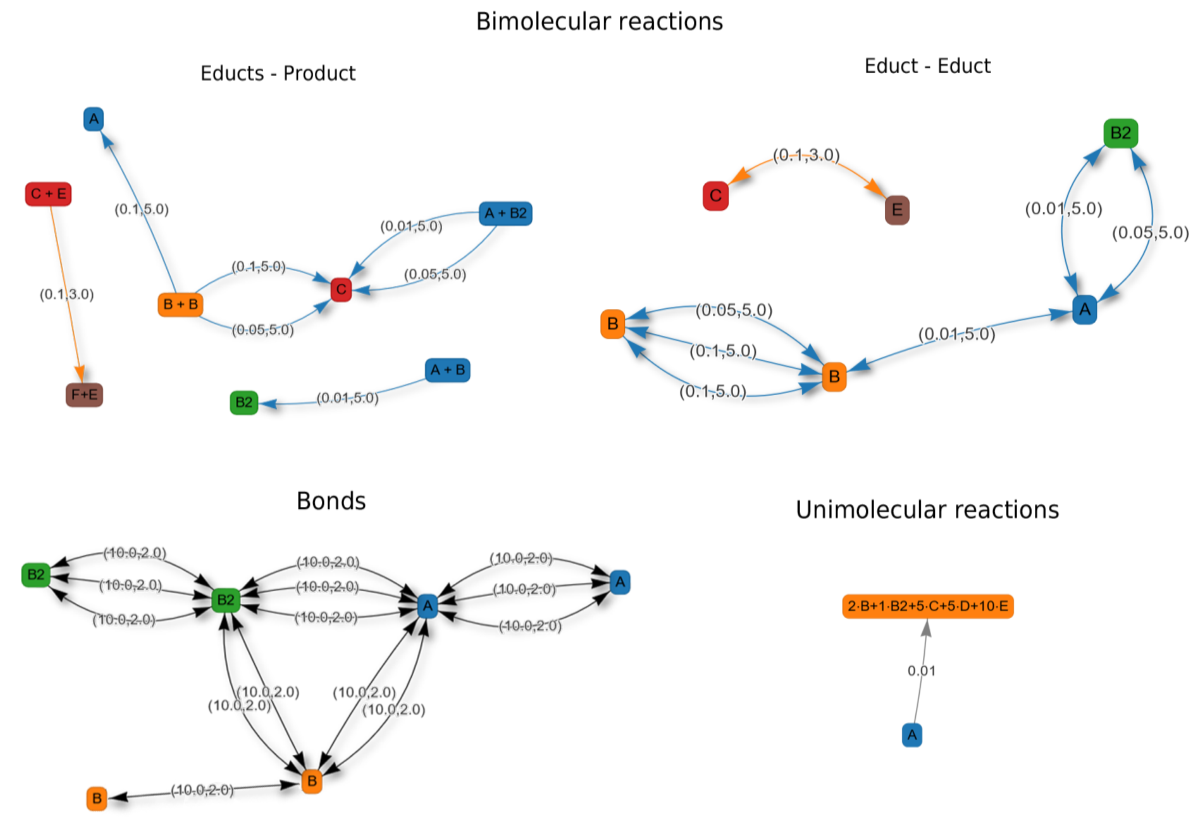

Figure S4. Examples of different reaction graphs. Related to Figure 7.
The ”Educts - Product” graph shows the reactants and their respective products as well as the various possible reaction paths. The reaction rate and the reaction radius are shown in round brackets. The ”Educt - Educt” graph only depicts the relationship between the different educts. The ”Bonds” graph shows the binding reactions between particle pairs.
