# Supplementary material for: The Brownian dynamics simulator PyRID for reacting and interacting particles written in Python
Source: Cell Rep Methods. 2025 Sep 18;5(10):101182. doi: 10.1016/j.crmeth.2025.101182 (PMC12570352; doi:10.1016/j.crmeth.2025.101182)
Supplement: Document S2. Article plus supplemental information [file mmc2.pdf]

Cell Reports Methods

Article

The Brownian dynamics simulator PyRID for reacting and interacting particles written in Python

Graphical abstract

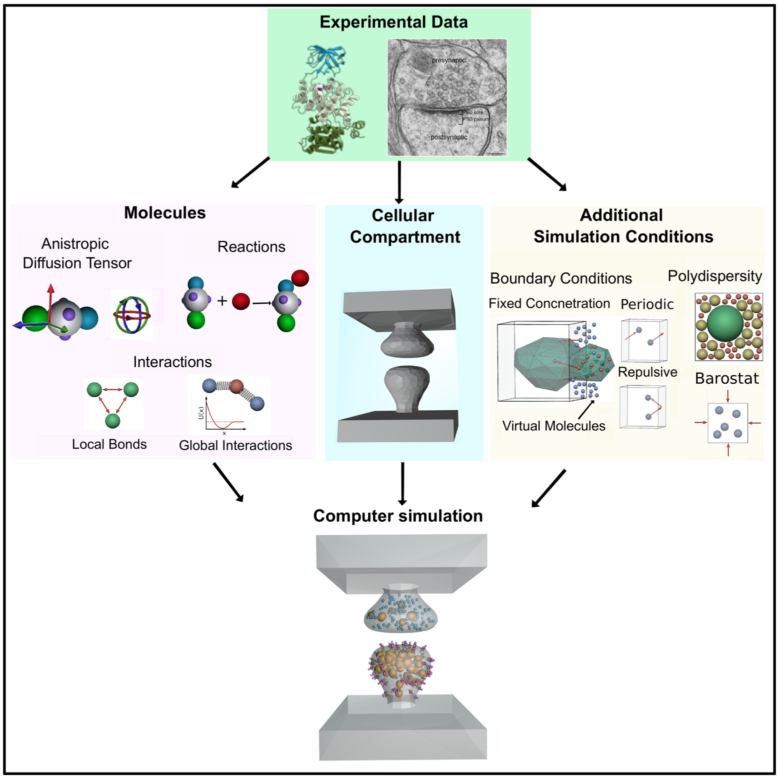

Highlights

- Efficient simulations of different complex biological molecular systems
- Interplay between membrane-associated and intra/extracellular processes
- Simulations of LLPS, nanodomains, and molecular reactions in biological systems
- Integrates multiple computational algorithms, ensuring a high degree of flexibility

Authors

Moritz Becker, Nahid Safari, Christian Tetzlaff

Correspondence

christian.tetzlaff@med.uni-goettingen.de

In brief

Becker et al. develop the Python-based software framework PyRID, which is optimized for the efficient simulation of complex biological molecular systems. PyRID supports diverse features, including multiple molecular reactions, membrane-associated processes, polydispersity, and dense molecular environments. PyRID is designed to facilitate accessibility, extensibility, and seamless integration into diverse research workflows.

Becker et al., 2025, Cell Reports Methods *5*, 101182
October 20, 2025 © 2025 The Author(s). Published by Elsevier Inc.
https://doi.org/10.1016/j.crmeth.2025.101182

CellPress

Cell Reports Methods

CellPress
OPEN ACCESS

Article

The Brownian dynamics simulator PyRID for reacting and interacting particles written in Python

Moritz Becker,[1] Nahid Safari,[1] and Christian Tetzlaff[1,2,*]

[1]Group of Computational Synaptic Physiology, Department of Neuro and Sensory Physiology, University Medical Center Göttingen, Göttingen, Germany

[2]Lead contact

*Correspondence: christian.tetzlaff@med.uni-goettingen.de

https://doi.org/10.1016/j.crmeth.2025.101182

MOTIVATION The inherent characteristics of biological molecular systems impose complex demands on computational simulations. These molecular systems are typically composed of densely packed substrates that consist of many differently sized molecules and proteins. Moreover, the cell membrane and organelles shape the space of molecule movement, including transmembrane proteins that move along the membrane and interact with proteins located in the intra- and extracellular spaces. The Python reaction interaction diffusion simulator (PyRID) addresses these high demands by integrating several computational algorithms while maintaining high performance. Based on the Python programming language, PyRID is designed to support extensibility, customization, and seamless integration into diverse software workflows.

SUMMARY

Recent advances in molecular biology have led to large-scale datasets providing new insights into the molecular organization of cells. To fully exploit their potential, computer simulations are essential to gain in-depth understanding of molecular principles. We developed the Python reaction interaction diffusion simulator (PyRID), a Python-based reaction-diffusion simulator designed for the efficient simulation of molecular biological systems. PyRID incorporates unimolecular and bimolecular reactions as well as pair interactions and simulation of individual interacting proteins to polydisperse molecular assemblies. It supports mesh-based compartments and surface diffusion of particles, enabling analyses of interactions between (trans) membrane proteins with intra- and extracellular proteins. Distinctively, PyRID uses hierarchical grids for polydisperse systems, supports rigid bead models, and calculates diffusion tensors internally. Validation against theoretical results and established models confirms PyRID's accuracy in reproducing key physical properties. PyRID is written entirely in Python, making it accessible to the broader scientific community, facilitating customization and integration into diverse research workflows.

INTRODUCTION

Cells are intricate structures composed of biomolecules interacting continuously to regulate cellular functions. Recent advancements in experimental techniques provide insights into the distribution, movements, and reactions of molecules within cells. However, observing the spatiotemporal interplay of many different molecule species is beyond reach, despite the great advances in technologies such as super-resolution microscopy or mass spectrometry. Computational modeling is essential for understanding molecular localization, movement, and interactions and to derive the underlying driving principles. Molecular dynamics simulations are well suited to integrate information of single molecules and to offer insights into the resulting dynamics of populations of molecules. Therefore, they are great tools that are used in different disciplines of physics, material sciences, or chemistry. However, investigating molecular processes in biological systems imparts special demands on the simulators; the intracellular as well as the extracellular fluid are, in general, highly polydisperse, meaning that they consist of many different molecule or protein species.[1,2] Proteins take up most of the intracellular space,[3,4] making a biological cell a dense molecular environment. The cell membrane as well as organelles shape the space of molecule movement[5,6]; transmembrane proteins like ion channels move along the membrane, interacting with proteins that are in the intracellular fluid.[7,8] To simulate physical

© 2025 The Author(s). Published by Elsevier Inc.
This is an open access article under the CC BY license (http://creativecommons.org/licenses/by/4.0/).

or chemical systems, often the thermodynamic assumption of an isolated system is being used to describe the system's environment. In a biological cell, each part continuously interchanges ions, proteins, etc. with its surroundings, making the formulation of an isolated system difficult, which has to be considered in the simulations. In recent years, simulators have been developed to meet several biological requirements. Building on these efforts, we developed PyRID (Python reaction interaction diffusion simulator), an easily extensible simulator designed specifically to include essential features for simulating molecular dynamics within biological systems, such as neurons. PyRID is a reaction-diffusion simulator that is completely written in the Python programming language and draws inspiration from other simulators, like MCell, Smoldyn, and ReaDDy.[9] PyRID is capable of simulating unimolecular and bimolecular reactions, pair interactions, mesh-based compartments, and surface diffusion. While the aforementioned simulators exhibit impressive performance, here, we will discuss their strengths and weaknesses to elucidate the rationale behind PyRID's development.

MCell (Monte Carlo Cell) is a stochastic simulator that is specialized in modeling reaction-diffusion processes within realistic three-dimensional cellular environments.[9–15] It employs a Monte Carlo approach to accurately simulate molecular diffusion and reactions and supports the use of triangulated meshes to represent complex cellular geometries, making it effective for simulating processes of, for instance, synaptic transmission, cellular signaling, and surface-bound reactions. Its integration with Blender (via CellBlender) allows users to construct detailed cellular geometries. MCell provides various boundary conditions, including periodic, repulsive, and fixed concentration boundaries. Additionally, it offers a Python interface and supports the BioNetGen reaction language, facilitating the integration of complex biochemical reaction networks. However, MCell lacks built-in support for force-based interactions, limiting its applicability to certain intracellular processes and in accounting for macromolecular crowding, as it primarily treats molecules as non-interacting point particles. Anisotropic translational diffusion is also not supported, and molecular structures are represented indirectly through internal state variables.

Smoldyn is a stochastic biochemical simulator similar to MCell that models each molecule as an individual particle in continuous space, providing nanometer-scale spatial resolution.[12,16,17] Smoldyn supports molecular diffusion, chemical reactions, molecule-surface interactions, and membrane-bound reactions, making it a versatile tool for modeling confined biochemical environments. Additionally, Smoldyn supports anisotropic translational diffusion and allows for distance-dependent interactions and movement based on user-defined equations. Its user interface is text based, with graphical output capabilities, and it offers APIs for C/C++ and Python, enhancing its versatility for integration into various workflows. However, Smoldyn only approximates excluded volume effects for spherical molecules and accounts only indirectly for molecular structures, impeding the simulation of macromolecular crowding.

ReaDDy (Reaction-Diffusion Dynamics) extends beyond diffusion by incorporating force-based interactions between particles, making it particularly useful for studying intracellular organization, bridging the gap between detailed molecular dynamics and large-scale reaction kinetics simulations.[13,18,19] It supports the definition of complex multi-particle structures through topology graphs and offers a wide range of interaction potentials, allowing for the modeling of molecular interactions, aggregation, and macromolecular crowding. In addition, ReaDDy supports a wide range of reactions, uses external potentials to confine particles, and supports periodic and repulsive boundary conditions. ReaDDy is implemented in C++ with Python bindings, providing flexibility in defining custom reaction rules and particle interactions. However, unlike MCell, ReaDDy does not support 3D meshes for modeling geometrically complex compartments and surfaces. Other limitations include the lack of rigid bead models for molecule representation, absence of anisotropic diffusion support, and the inability to implement fixed concentration boundary conditions. Additionally, polydispersity can lead to significant performance drops in simulations.

In addition to the biological systems simulators discussed above, several general-purpose molecular simulation frameworks have gained widespread adoption in computational chemistry and physics.[20–22] For instance, GROMACS and LAMMPS are molecular dynamics engines that are highly optimized in simulating large-scale atomic systems with detailed force fields. These simulators are extensively used for modeling molecular interactions, folding, and dynamics at atomistic resolution, but they are less suited for modeling mesoscopic reaction-diffusion systems or complex cellular geometries. NERDSS (Non-Equilibrium Reaction-Diffusion Self-assembly Simulator), on the other hand, is a recent simulator that bridges this gap by enabling reaction-diffusion simulations with rule-based modeling of molecular assembly. While these tools are powerful in their respective domains, they often lack built-in support for key biological features, such as surface-bound diffusion or realistic cellular topologies, which are addressed by PyRID.

In order to have a simulator integrating a wide spectrum of features desired for investigating biological systems, we developed PyRID, which incorporates various tools from other simulators. In addition, as the rapid development in the research of biological systems implies the need to easily adapt the source code of a simulator by individual researchers, different from the simulators discussed above, PyRID is written entirely in Python, a programming language known for its readability and easy accessibility. As a core component, in PyRID, a molecule can be described by one or more rigidly connected beads of arbitrary spatial configuration with corresponding anisotropic translational and rotational diffusion. On the surface of each bead, several specific patches (represented as particles) can be defined that determine the interaction of the molecule with others, allowing multivalent protein-protein interactions. As each molecule can have a structure that is defined by beads of different sizes, using a hierarchical grid data structure, PyRID supports an efficient simulation of polydisperse systems. Triangulated mesh geometries can be formulated for examining the influence of different cell geometries on molecular organization. Furthermore, the movement of molecules can be constrained to these geometries, enabling the investigation of transmembrane protein dynamics, including their interaction with intra- or extracellular proteins. PyRID allows the consideration of boundary conditions with fixed concentrations of molecules to emulate the behavior of the simulated

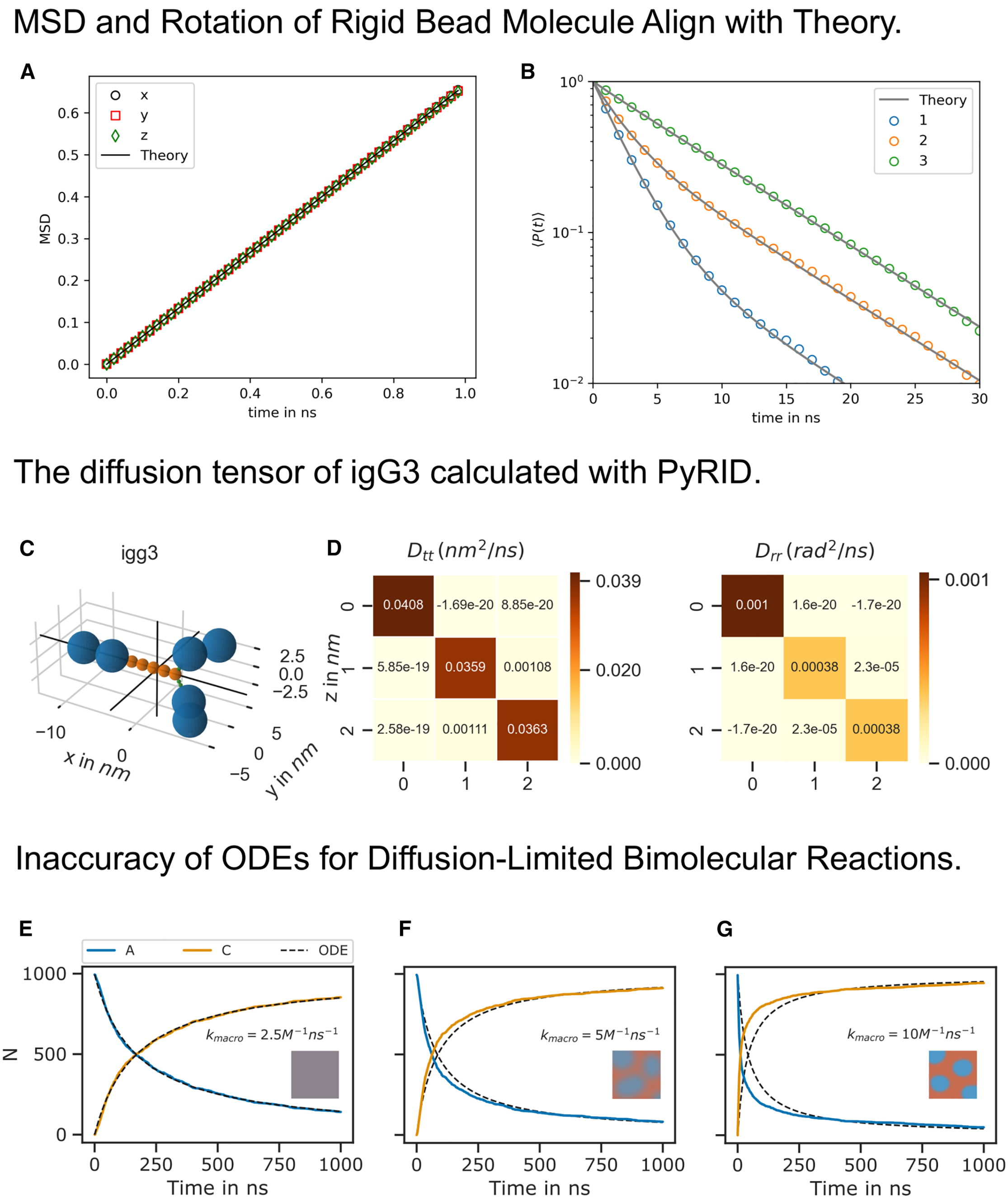

Figure 1. Implementation of diffusion-dependent processes in PyRID

(A and B) MSD and rotational relaxation times of a rigid bead molecule matches the theoretical prediction. (A) MSD of the rigid bead molecule computed with PyRID. The displacement in each dimension (colored markers) is in very good agreement with the theory (black line).

(B) The rotational relaxation of the rigid bead molecule is also in close agreement with theory (gray lines) for each of the rotation axes (colored markers).

(C and D) The diffusion tensor of igG3 calculated with PyRID.

(C) Rigid bead molecule representation of igG3. The origin of the coordinate system is set to the center of diffusion of the igG3 rigid bead model (bold black lines).

(D) Translational and rotational diffusion tensor of igG3.

(E–G) Diffusion-limited bi-molecular reactions are not accurately described by ODEs. Shown is the minimal system $A + B \xrightarrow{k_1} C$ with $R_{react} = 4.5\,nm$ and $\sigma_A = 3\,nm$, $\sigma_B = 4.5\,nm$, $\sigma_C = 3.12\,nm$. The same system has been used for validation of ReaDDy.[13] The ODE approach to the description of the reaction kinetics assumes a well-mixed system. If the reaction rate is small, then the system has enough time to equilibrate in between reactions, and the ODE approach (black dotted lines) and the particle-based stochastic simulation algorithm approach (colored lines) match (E). However, with increasing reaction

(legend continued on next page)

system as a compartment in a continuous interchange with the rest of the cell. PyRID also exhibits high accuracy in simulating various types of reactions and is readily modifiable and expandable by Python programmers. Moreover, PyRID employs Numba just-in-time (JIT) compilation to achieve efficient performance. These features, including the use of hierarchical grids for polydisperse systems, support for rigid bead models, and the capability to calculate diffusion tensors within the simulator, clearly set PyRID apart from existing simulators. The complete PyRID code, along with extensive online documentation and examples, is available on the GitHub repository:

Code: https://github.com/MoritzB90/PyRID;

Documentation: https://moritzb90.github.io/PyRID_doc/.

In the STAR Methods, we introduce the main features and rationale of PyRID. Under results, we provide different examples from computational chemistry and biology for validation and show PyRID's performance together with some use cases.

RESULTS

Anisotropic diffusion

We verified the accuracy of our translational and rotational diffusion algorithms using the example from Ilie et al.,[23] where the diffusion tensors are not molecule specific. We compared the mean squared displacement (MSD) and rotational time correlation with theoretical predictions, using a multi-exponential decay function for the latter.[24] The MSD is given by

$$MSD(t) = \langle |x(t+\Delta t) - x(t)|^2 \rangle. \qquad \text{(Equation 1)}$$

The rotational time correlation function is given by

$$P(t) = \frac{3}{2}\langle (n(t+\Delta t)n(t))^2 \rangle - \frac{1}{2}, \qquad \text{(Equation 2)}$$

where $n(t)$ is some unitary vector that describes the current orientation of the molecule at time point t. Figures 1A and 1B show the comparison between simulation results and theoretical prediction.

Diffusion tensor of the igG3 protein

The methods described to compute diffusion tensors were previously only available in Hydro++, but we have now incorporated them directly into PyRID for efficient setup of rigid bead molecule systems. We tested our implementation using the igG3 protein model (Figures 1C and 1D) provided in the Hydro++ documentation and obtained results that are in good agreement with Hydro++ at up to 4 decimal places.

Choosing the right reaction rate and radius

In molecular simulations, accurately choosing reaction parameters, such as reaction rate (k) and reaction radius (R_{react}), is crucial for capturing realistic system behavior. Here, it is important to choose R_{react} carefully to avoid reactions with non-nearest neighbors in dense systems.

However, R_{react} should also not be smaller than the average change in the distance between molecules, which is given by $\lambda_{AB} = \sqrt{4(D_A^t + D_B^t)\Delta t}$, where D_A^t and D_B^t are the translational diffusion constants of two molecular species A and B. Otherwise, a molecule might pass many reaction partners in between two time steps where the bi-molecular reactions are not evaluated.[25] Alternatively, as proposed by Schöneberg and Noé,[18] one can use the electrostatic interaction length scale to define R_{react}.

A description of the reaction kinetics in terms of a system of differential equations assumes a well-mixed system. Therefore, the simulation results are also only directly comparable with the ODE approach if the reactions are reaction rate limited, not diffusion limited, so that the system has enough time to equilibrate in between reactions. In a simple $A+B\rightarrow C$ reaction with diffusion-limited kinetics, the local concentration of educts only decreases gradually, as mixing with products by diffusion is slow in comparison to the microscopic reaction rate. As a consequence, the probability of encounter for the remaining educts also only decreases slowly. Educts and products are heterogeneously distributed; the system is not well stirred. In contrast, in a well-stirred system, the concentration of educts globally and locally decreases, reducing the probability of educt encounters. Since the assumption of a well-stirred system is no longer valid for diffusion-limited kinetics, the reaction kinetics in the ODE approach are slowed down compared to the stochastic particle-based simulation (Figures 1E–1G).

The reaction rate determining the probability that a reaction occurs within a given time step in particle-based simulations is not the same as the reaction rate in the ODE approach. In line with Schöneberg and Noé,[18] we refer to the latter as the macroscopic rate and other as the microscopic rate. To compare the particle-based simulations to the ODE approach, the microscopic reaction rates need to be converted to the corresponding macroscopic rates or vice versa, which can be done by taking into account the reaction radius and the diffusion constants of the reacting species.

Given a reaction radius R_{react}, we would like to know at what reaction rate k a simulation would match an experimentally measured macroscopic reaction rate k_{macro}. For two non-interacting molecule species A and B with translational diffusion constants D_A^t and D_B^t and $\lambda_{AB} \ll R_{\text{react}}$, k_{macro} is given by[25]

$$k_{macro} = 4\pi(D_A^t + D_B^t)\left[R_{react} - \sqrt{\frac{D_A^t + D_B^t}{k}}\,tanh\left(R_{react}\sqrt{\frac{k}{D_A^t + D_B^t}}\right)\right]. \qquad \text{(Equation 3)}$$

Equation 3 can be solved numerically for k.

rates (F and G), the system becomes heterogeneous over time, resulting in regions of varying educt concentrations. Initially, stochastic simulations exhibit faster kinetics than ODE predictions (F and G). However, when a critical mass of educts has reacted, slow diffusion causes kinetics to deviate from ODE predictions, necessitating two exponential functions instead of one. The slowdown effect is especially prominent (F and G) at around 500 ns. The reaction kinetics are therefore better described by two exponential functions instead of one.

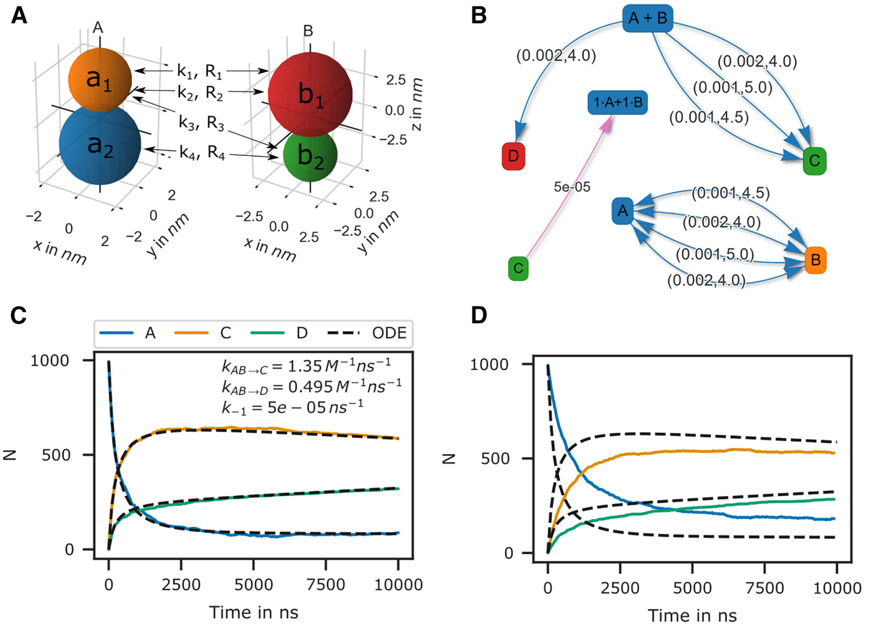

Figure 2. Bi-molecular reaction between two rigid bead molecules

(A) Depiction of the two rigid bead molecules and the different reactions defined on their respective particles/beads.

(B) Reaction graphs showing the different reaction paths for the fusion reactions and the fission reaction. The bottom right graph depicts the different reaction paths between the two educts A and B without specifying the products. In total, there are 4 paths.

(C) If not accounting for any repulsive interaction between molecules A and B, then the simulation results are in good agreement with the ODE description.

(D) If we account for the excluded volume of the molecules by a repulsive interaction potential, then the results of the particle simulation and the ODE no longer match.

PyRID has a built-in method to calculate the microscopic reaction rate k and the macroscopic reaction rate k_{macro} from Equation 3.

Bi-molecular reactions between rigid bead molecules

The use of single particles to represent complex molecules neglects their structure and potential for bi-molecular reactions via different sites. PyRID addresses this limitation by enabling the simulation of reactions between complex molecules with different reaction sites represented by beads/patches in the rigid bead molecule's topology. Bi-molecular reactions can be defined on particles or molecules but in the latter require linkage to a particle type pair for distance computation. Successful reactions on molecule types occur if the two particles are within the reaction distance. As an example of bi-molecular reactions, we consider molecules A and B that are represented by two beads each a_1, a_2 and b_1, b_2, and reaction $A + B \rightarrow C$ as well as $A + B \rightarrow D$. We now may define reactions for different pair permutations of the available beads as follows:

$$\begin{aligned} A(a_1) + B(b_1) &\xrightarrow{k_1 R_1} C, \\ A(a_1) + B(b_1) &\xrightarrow{k_2 R_2} D, \\ A(a_1) + B(b_2) &\xrightarrow{k_3 R_3} C, \\ A(a_2) + B(b_2) &\xrightarrow{k_4 R_4} C, \\ C &\xrightarrow{k_{-1} R_{-1}} A + B, \end{aligned} \qquad \text{(Equation 4)}$$

where k_i are the microscopic reaction rates and R_i the reaction radii. For better visualization, see Figures 2A and 2B. Whereas for the particle pairs (a_1, b_2) and (a_2, b_2) only one reaction pathway is defined, for the particle pair (a_1, b_1), a second reaction path has been defined for the fusion of molecules A and B to molecule D. We may also describe this system in terms of a system of ODEs:

$$\begin{aligned} \frac{dA}{dt} &= -\left(k^1_{macro} + k^3_{macro} + k^4_{macro}\right)AB - k^2_{macro}AB + k^{-1}_{macro}C, \\ \frac{dB}{dt} &= -\left(k^1_{macro} + k^3_{macro} + k^4_{macro}\right)AB - k^2_{macro}AB + k^{-1}_{macro}C, \\ \frac{dC}{dt} &= -\left(k^1_{macro} + k^3_{macro} + k^4_{macro}\right)AB + k^{-1}_{macro}C, \\ \frac{dD}{dt} &= k^2_{macro}AB. \end{aligned} \qquad \text{(Equation 5)}$$

The macroscopic rate constants k_{macro} can be calculated from Equation 3. For complex molecules, however, Equation 3 is inadequate for calculating the macroscopic rate constants, as it only accounts for the translational diffusion constant of the molecule center and not the rotational motion. For simple molecules such as the bead in our example, the Brownian dynamics simulation results align closely with the ODE formulation (Figure 2C), indicating a motion similar to that of a single spherical particle. However, for more complex molecules with anisotropic reaction volumes, a deviation from this approximation is expected. Although the rigid bead model may not seem particularly advantageous over other stochastic simulation algorithms for the above example, it already becomes useful when considering excluded volume effects. By introducing repulsive interactions between the beads, we observe differences in reaction kinetics when compared to the ODE solution (Figure 2D).

Hard-sphere fluid

For validation purposes, a hard-sphere fluid is advantageous due to the availability of analytical expressions for the radial

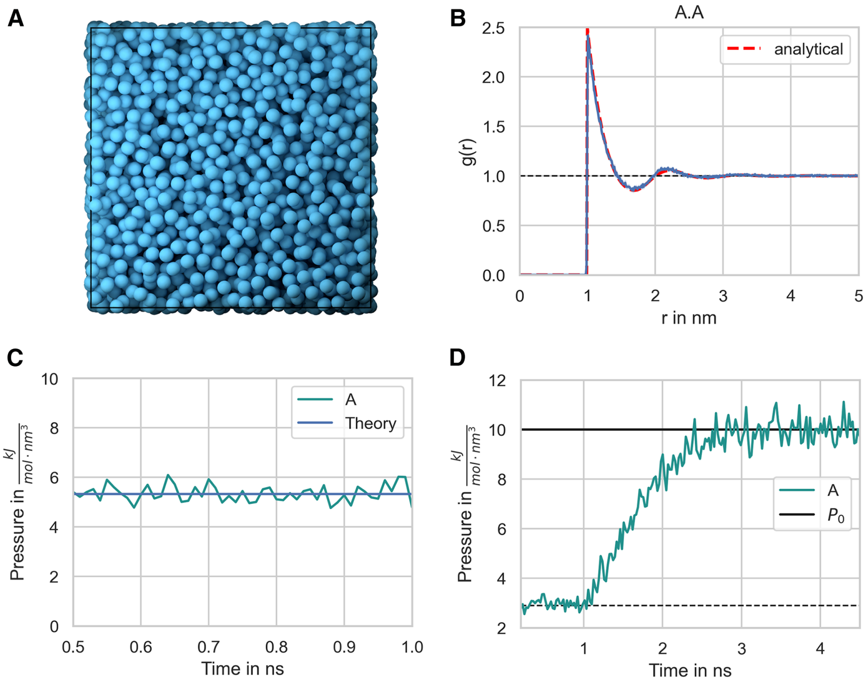

Figure 3. Hard-sphere radial distribution function

(A) The system is set up with a packing fraction of $\eta = 0.3$. The particle diameter is set to 1 nm, and pair interactions occur via a harmonic repulsive potential.

(B) The resulting radial distribution function (blue line) is in close agreement with theoretical prediction (red line).

(C) The pressure of the hard-sphere fluid obtained from simulations is also in close agreement with theory[26] (blue line).

(D) A hard-sphere fluid NPT ensemble simulation. From time 0.5 ns, the Berendsen barostat is activated and drives the system to the target pressure. $P_0 = 10\,kJ/(mol\,nm^3) = 16.6\,MPa = 166\,bar$. The simulation was performed with a diffusion coefficient of 0.0859 nm^2/ns and a time step of 0.1 ns, ensuring both stability and accuracy in the results.

distribution function and pressure. In our simulations, we approximate hard sphere interactions using a harmonic repulsive potential rather than a step function repulsion. While a true hard sphere interaction would feature an infinite energy barrier at a fixed distance, a harmonic repulsion provides a continuous force that, with proper parameterization, effectively prevents particle overlap while closely mimicking the behavior of a hard sphere system. This approach enables stable simulations while still reproducing key structural and thermodynamic properties of a hard-sphere fluid (Figure 3A).

Radial distribution function

In Figure 3B, the radial distribution function is shown for a hard sphere-fluid simulated with a harmonic repulsive interaction potential and a sphere diameter of 1 nm. The simulation result agrees well with an analytical expression for the hard sphere radial distribution function from Trokhymchuk et al.[26]

Pressure

A hard-sphere fluid can serve as a reliable means of validating pressure calculations. The pressure can be expressed analytically in terms of the radial distribution function at contact and the second virial coefficient,[27] with the Percus-Yevick equation offering an approximation for the radial distribution function at contact.[28] The simulation results for the pressure of a hard-sphere fluid closely match theoretical expectations (Figure 3C), and the system reaches the target pressure when utilizing the Berendsen barostat (Figure 3D).

Benchmarks

Polydispersity

As mentioned under STAR Methods, PyRID uses a hierarchical grid approach to efficiently handle polydispersity. As a test, a two-component system is used. Both components consist of a single particle. Component A has a radius of 10 nm, and component B has a radius of 2.5 nm. The simulation box measures 75 × 75 × 75 nm. The simulation volume is densely packed with both components so that we reach a volume fraction of 52%. The simulation ran for 10,000 steps. When not using the hierarchical grid approach but the classical linked cell list algorithm, PyRID only reaches about 80,000 particle updates per second (pu/s) on average (Figure 4A). However, when using the hierarchical grid, more than 500,000 pu/s are reached. If instead of the two-component system we only simulate a one-component system, PyRID also only reaches about 500,000 pu/s. Thereby, PyRID performs similarly independent of whether the system is mono- or polydisperse.

Molecular interactions and reactions

We used a benchmark test as in ReaDDy,[13] which consisted of three molecule types, A, B, and C, with radii of 1.5, 3.0, and 3.12 nm, respectively. The molecules interacted via a harmonic repulsive potential with a force constant k = 10 kJ/mol, and the interaction distance σ was given by the radii of the interacting molecule pair:

$$U(r) = \begin{cases} \frac{k}{2}(r-\sigma)^2, \text{if } r \le \sigma \\ 0, \quad \text{otherwise} \end{cases}. \quad \text{(Equation 6)}$$

The system also included a fusion reaction $A + B \xrightarrow{k_1} C$ with a rate constant $k_1 = 0.001\ ns^{-1}$ and a reaction radius $R_{react} = 4.5$ nm as well as a fission reaction with a rate constant $k_{-1} = 5 \times 10^{-5}\ ns^{-1}$ and a dissociation radius equal to R_{react}. The benchmark was carried out for different values of the total initial molecule number N_{tot}, with $N_A = N_{tot}/4$, $N_B = N_{tot}/4$, and $N_C = N_{tot}/2$, while keeping the number density constant at $\rho_{tot} = 0.00341\ nm^{-3}$. Simulations were performed for 300 ns with an integration time step of 0.1ns.

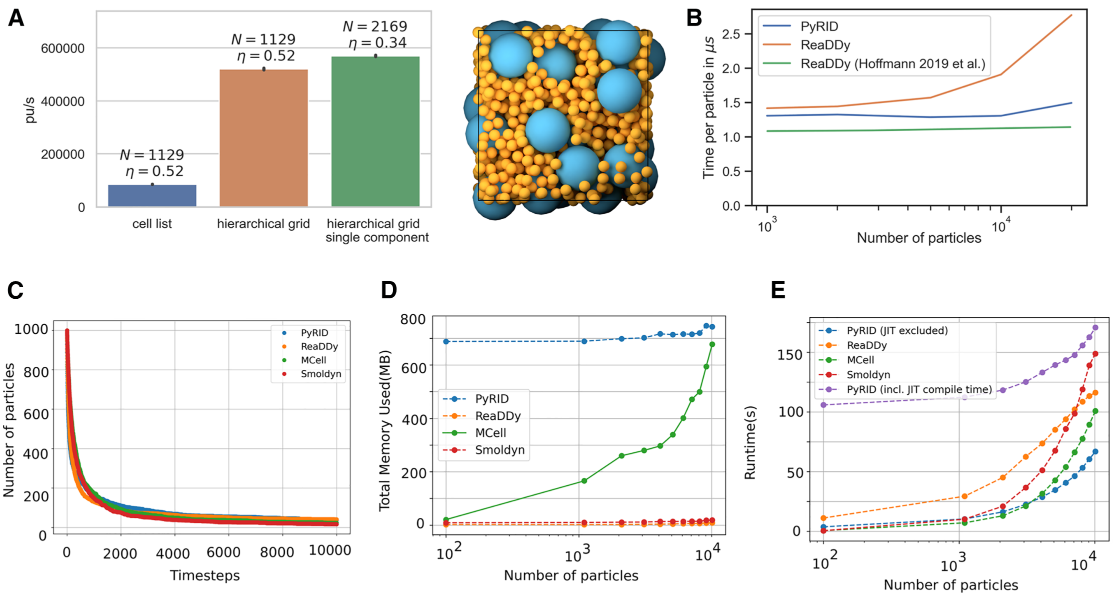

Figure 4. Performance tests and benchmarking

(A) Performance hierarchical grid.

(B) Performance comparison between PyRID and ReaDDy. On a benchmark system with an Intel Core i5-9300H with 2.4 GHz and 24 GB DDR4 RAM, PyRID (blue line) outperforms ReaDDy (yellow). However, Hoffmann et al.[13] obtained a better performance and especially scaling for ReaDDy on a different machine with an Intel Core i7 6850K processor at 3.8 GHz and 32 GB DDR4 RAM (green line).

(C) Validation of the reaction dynamics for the model $A+B \rightarrow C$ across different particle-based simulators. The temporal evolution of species A, B, and C is similar between the simulators, confirming the correctness and consistency of the implemented reaction mechanism.

(D) Comparison of memory usage across PyRID, ReaDDy, MCell, and Smoldyn for the reaction $A+B \rightarrow C$, simulated for increasing numbers of particles. PyRID shows a higher baseline memory footprint, attributed to its broader set of features, but a slower increase in required memory for larger systems.

(E) Runtime performance comparison for the reaction $A+B \rightarrow C$ using PyRID, ReaDDy, MCell, and Smoldyn. Two curves are shown for PyRID; one includes the Numba JIT compilation time, and the other excludes it. When compilation time is included, MCell and Smoldyn exhibit the fastest runtimes, while PyRID remains competitive despite supporting extended features. When compilation time is excluded, PyRID achieves the best runtime performance among all simulators.

The performance test results showed that PyRID outperformed ReaDDy for this benchmark test (Figure 4B, blue vs. orange curve). For particle numbers between 1,000 and 10,000, the computation time per particle update stayed approximately constant at 1.25 μs, which corresponded to about 800,000 pu/s. For particle numbers above 10,000, the performance dropped slightly. The benchmark test was performed on a machine with an Intel Core i5-9300H with 2.4 GHz and 24 GB DDR4 RAM. The performance test for ReaDDy was carried out on the sequential kernel (parallel kernels were even lower in performance), and the results showed that ReaDDy scaled less linearly for large particle numbers than PyRID.

The reason why ReaDDy's scaling behavior for large particle numbers was much less linear in the benchmark test and why multi-threading led to a performance loss was not clear, but it was speculated that ReaDDy might have been compiled differently in the benchmark system used in Hoffmann et al.[13] than the binaries used in this study (Figure 4B. green curve). Nonetheless, the benchmark test showed that PyRID's performance was comparable to ReaDDy, and in certain situations, PyRID could even outperform ReaDDy. For a system with 10,000 particles, PyRID was able to perform at approximately 80 it/s, corresponding to approximately 7×10^6 it/day. Therefore, at an integration time step of 1 ns, approximately 7 ms per day could be simulated on a medium machine.

Performance evaluation

To evaluate the computational efficiency of the particle-based simulators employed in this study—PyRID, ReaDDy, MCell, and Smoldyn—we benchmarked their performance using a simple bimolecular reaction system, $A+B \rightarrow C$, simulated inside a cubic compartment. Figure 4C demonstrates that all simulators produce consistent reaction dynamics, validating their accuracy and making them suitable for comparative and complementary use depending on the research context. The simulations were run for increasing particle counts, and for each case, we measured both peak memory usage (in megabytes) and runtime (in seconds). The results are summarized in Figure 4D for memory usage and Figure 4E for runtime. The runtime comparison revealed that Smoldyn and MCell were the fastest simulators, with significantly lower execution times across all particle numbers. PyRID performed comparably well, while ReaDDy consistently exhibited the longest runtimes. In terms of memory usage, PyRID showed the highest values among the four simulators. However, this higher usage is not excessive and remains within acceptable limits. Importantly, PyRID supports a broader range of features, —such as flexible mesh support, reaction zones, and hybrid dynamics, which likely accounts for its memory footprint.

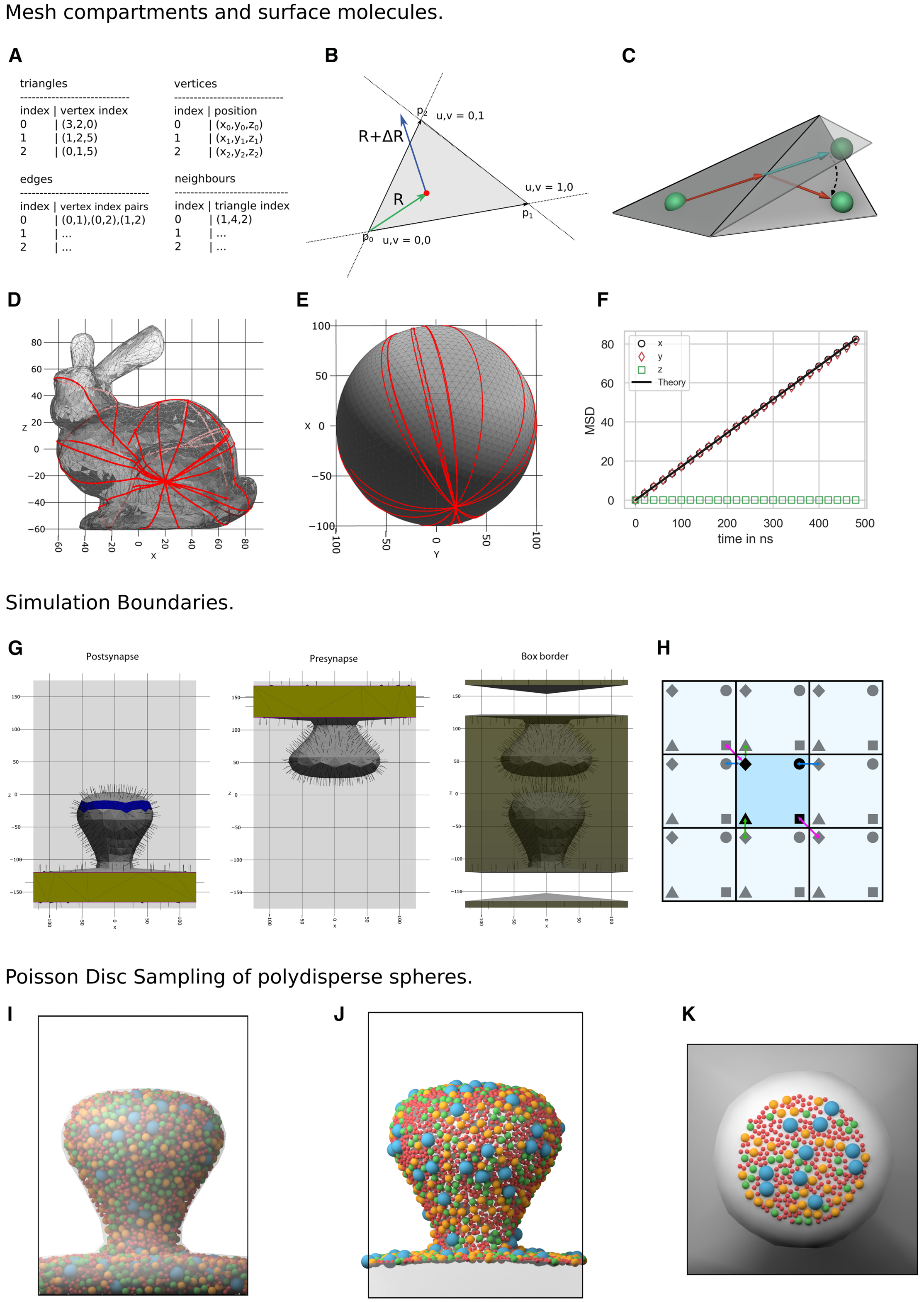

(legend on next page)

DISCUSSION

In this article, we have presented PyRID, a Python-based Brownian dynamics simulator designed for interacting and reacting particles.

PyRID incorporates multiple features from existing tools, including rigid bead models for reduced particle simulation, accurate diffusion motion via experimental and theoretical diffusion tensors, and patches on bead model surfaces for multivalent protein-protein interactions. The simulator also considers polydispersity of particle sizes through a hierarchical grid data structure, supports triangulated mesh geometries to account for compartmentalized 3D environments and fixed concentration boundary conditions for simulating subregions within larger systems, and allows the implementation of a multitude of uni- and bimolecular reactions as well as surface diffusion. PyRID is easily modifiable and expandable by Python programmers and utilizes Numba JIT compilation for efficient performance, achieving results comparable to ReaDDy.

Limitations of the study

PyRID does not currently incorporate hydrodynamic interactions between molecules due to the computational cost associated with recalculating the 6 N $\times$ 6 N diffusion tensor at every time step, which would render large-scale simulations impractical. However, future work may explore computationally efficient approximations, such as the Rotne-Prager-Yamakawa tensor or Stokesian dynamics, which have been successfully applied in related contexts.[29–32] Implementing these approaches in PyRID could enable simulations that capture hydrodynamic effects while maintaining computational feasibility. A discussion on this topic in the context of many-particle simulations can be found in Geyer and Winter.[33] Unlike ReaDDy, PyRID currently supports only pairwise interaction potentials for bond constraints and does not yet include angular constraints for particle triplets or torsion potentials for particle quadruplets. While such constraints are necessary for accurately modeling sub-protein structures, PyRID is primarily designed to efficiently handle rigid bead models and pairwise interactions. Implementing angular constraints and torsion potentials is a natural next step in PyRID's development and is planned for future updates. For users requiring these features, ReaDDy offers an alternative with built-in support for angular constraints and an algorithm that ensures detailed balance for reversible reactions, improving accuracy in dense systems.[19]

PyRID is a powerful tool for particle-based reaction diffusion simulations with pair interactions, but it currently cannot simulate processes that occur over long timescales of several seconds or minutes. This limitation is significant because many cellular processes, such as signaling, self-assembly of clathrin, and protein trafficking, operate on such timescales. Other similar tools, like ReaDDy,[13,18] also lack the ability to simulate on long timescales. While MCell[9] and Smoldyn[12] allow for longer simulations, they cannot resolve molecular structure or simulate protein binding and assembly. However, alternative reaction rate-based approaches that consider molecule structure, binding, and diffusion have been developed. A prominent example is NERDSS,[34] which is able to resolve fast binding reactions as well as processes on large timescales and spatial scales. NERDSS employs rigid body representations of molecules, like PyRID, and utilizes rejection sampling to account for excluded volume effects. However, unlike PyRID, NERDSS does not incorporate energy interaction functions, instead relying on predefined "snapping" of molecules during binding reactions to facilitate efficient simulation of assembly processes. Despite the efficiency of NERDSS in simulating assembly processes, it has limitations in predicting the structure of protein assemblies due to the predefined form of resulting assemblies. Furthermore, the lack of orientation dependence in binding reactions can lead to unrealistic events, unlike PyRID, which considers orientation dependence in its construction. The use of NERDSS for describing assembly and disassembly processes relies on reaction rates measured by experimental or molecular dynamics simulation techniques. However, accurately modeling such

Figure 5. Compartments, boundaries, and polydispersity in PyRID

(A–F) Mesh compartments and surface molecules.

(A) PyRID uses triangulated meshes to represent compartments. These are kept in a shared vertex mesh data structure.[52] In addition, for neighbor search, two arrays are used that hold, for each triangle, the vertex indices of the three triangle edges and the triangle indices of the three triangle neighbors.

(B) Triangle vertices are ordered counterclockwise, as are edges. Efficient algorithms based on barycentric triangle coordinates are used to check whether a point lies within a triangle or whether a displacement vector intersects a triangle edge.

(C) Visualization of mesh surface ray marching. If a molecule (green sphere) crosses a triangle edge, its displacement vector is advanced to the corresponding edge and then rotated into the plane of the neighboring triangle.

(D and E) By the ray marching method described in the text, molecules follow geodesic paths on the mesh surface.

(F) The MSD of diffusing surface molecules is in agreement with theory, which is in 2 dimensions $MSD = 4Dt$ (here, $D = 43\ nm^2/\mu s$).

(G and H) Simulation boundaries.

(G) Left, middle: when a compartment intersects with the simulation box, the intersecting triangles are assigned to a transparent class (yellow), as are the corresponding edges that intersect with the boundary (purple lines). If boundary conditions are set to "fixed concentration," then transparent triangles and edges act as absorbing boundaries but, in addition, release new molecules into the simulation volume. Furthermore, the user can combine mesh triangles into face groups, which enable the user, for instance, to distribute molecules on specific regions of the mesh surface (blue area on the left). Right: if mesh compartments intersect the boundary of the simulation box, then the remaining part of the box boundary must also be represented by a triangulated mesh.

(H) For periodic boundary conditions, PyRID follows the minimal image convention; i.e., a particle (black marker) only interacts (colored arrows) with the closest image (gray marker) of the other particles in the system.

(I–K) Poisson disc sampling of polydisperse spheres.

(I) Example distribution for three differently sized particle types confined to the volume of a mesh compartment.

(J) Poisson disc sampling for surface molecules.

(K) Poisson disc sampling for surface molecules being restricted to a surface region that is defined by a triangle face group.

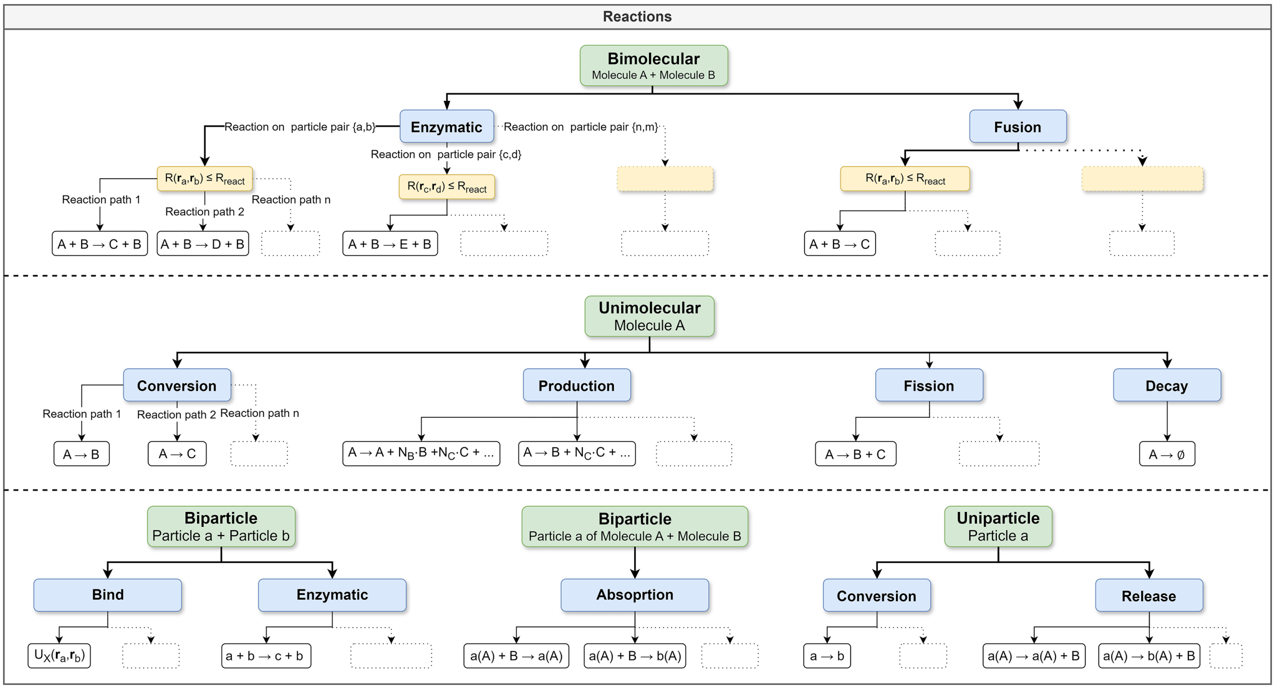

Figure 6. This tree graph provides an overview of the supported bi- and unimolecular reactions

A distinction is made between reactions that affect the molecules (uppercase) and those affecting the particles (lowercase). Both bimolecular and biparticular reactions are analyzed on the basis of particle pair distances $R(r_i, r_j)$, since only these are calculated during the simulation and not, for example, the distances between the molecule's geometric centers. Multiple reactions can be defined for the same particle pair, particle, or molecule (e.g., a fusion and an enzymatic reaction), and multiple reaction paths can be defined for each of these reaction types, leading to various possible reaction outcomes or products.

processes using PyRID is also challenging, as it requires precise specification of energy functions governing binding interactions, which can be even more difficult to estimate than reaction rates. Additionally, NERDSS lacks interaction forces, rendering it unable to compute physical properties derived from energy functions or handle flexible chains of beads or molecules. Nonetheless, rate-based approaches like NERDSS are promising for studying complex assembly kinetics and could complement PyRID, which can enable rigid body assembly growth in principle. However, the inclusion of energy functions imposes an upper limit on the integration time step, which can be slightly increased by approximating intermolecular interaction energy functions. To address this computational bottleneck, parallelization is necessary.

For simulating very large systems, molecular dynamics tools like LAMMPS often offer parallel implementations of their algorithms using the message passing interface standard. However, the benefits of parallelization are limited to large systems, as message passing can become a bottleneck, and may not result in significant speedup. The scalability of parallelization depends on the size of the system being simulated, and careful consideration is required when selecting the appropriate parallelization strategy for molecular dynamics simulations.

For simulating intermediate-sized systems containing 10,000–100,000 particles, GPU-based algorithms offer significant performance advantages over CPU-based methods. For instance, tools like HooMD,[35] optimized for GPUs, can achieve speedups of up to two orders of magnitude compared to a single CPU and over one order of magnitude compared to a modern multi-core CPU. By leveraging the power of GPUs, particle-based reaction-diffusion simulations could be conducted on timescales of minutes. However, the challenge remains in adapting the necessary algorithms and data structures to effectively run on GPUs. Additionally, machine learning has shown promise in applications such as coarse graining and molecular kinetics,[36,37] highlighting the potential of emerging technologies to advance simulations. In the case of PyRID, while Python is typically slower than compiled languages like C++, the tool mitigates this limitation by leveraging Numba's JIT compilation, which achieves speeds comparable to C or C++ for critical functions. This approach ensures computational efficiency without compromising the flexibility of Python. Although rewriting the core algorithms in C++ could offer further speed improvements, the existing JIT implementation already provides substantial performance gains, particularly for small- to medium-sized models. Looking ahead, we recognize the potential benefits of GPU acceleration for highly parallelizable tasks. While adapting PyRID's algorithms for GPU architectures presents unique challenges, exploring GPU acceleration represents a promising avenue for improving the computational efficiency of more complex simulations. We plan to investigate this in future updates to ensure that PyRID remains competitive with other GPU-accelerated tools like ARBD.[38,39]

PyRID does not currently include a built-in feature for directly incorporating experimental or bioinformatics data. This is an important focus for future development, as such a feature will simplify the initialization of models or the refinement of model

CellPress
OPEN ACCESS

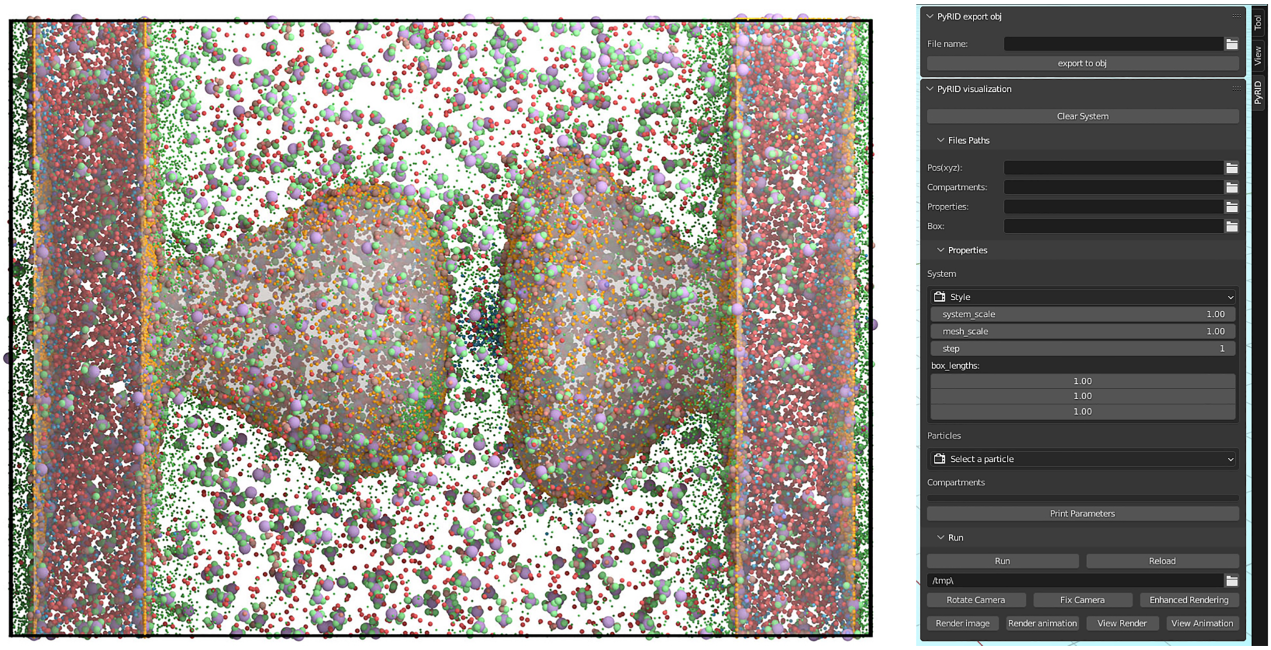

Figure 7. Visualization of molecule trajectories with the PyRIDs Blender add-on

Left: example visualization with 50.000 particles. Right: GUI of the Blender add-on.

parameters. By integrating experimental data with computational models, PyRID will be better equipped to capture the complexities of biological systems and improve the accuracy of its predictions. We view this as a critical step toward making PyRID an even more powerful tool for simulating complex biological phenomena.

Currently, PyRID does not provide built-in tools for mapping coarse-grained models to their corresponding atomistic representations. As a result, tasks such as structure-based coarse-graining and force matching must be carried out using external tools or custom scripts. A wide range of established methodologies and tools exist in the literature to support these procedures, which can be integrated with PyRID as needed.[40–42]

RESOURCE AVAILABILITY

Lead contact

Requests for further information and resources should be directed to and will be fulfilled by the lead contact, Christian Tetzlaff (christian.tetzlaff@med.uni-goettingen.de).

Materials availability

This study did not generate new unique reagents.

Data and code availability

- All data reported in this paper will be shared by the lead contact upon request.
- All original code is publicly available at https://doi.org/10.5281/zenodo.16946717 as of the date of publication and is also available at https://github.com/MoritzB90/PyRID.
- Any additional information required to reanalyze the data reported in this paper is available from the lead contact upon request.

ACKNOWLEDGMENTS

This work was funded by the German Research Foundation (DFG) via the CRC1286, projects C1 and Z1.

AUTHOR CONTRIBUTIONS

Conceptualization, M.B. and C.T.; methodology, M.B. and N.S.; investigation, M.B. and N.S.; writing – original draft, M.B., N.S., and C.T.; writing – review and editing, M.B., N.S., and C.T.; funding acquisition, C.T.; resources, C.T.; supervision, C.T.

DECLARATION OF INTERESTS

The authors declare no competing interests.

STAR★METHODS

Detailed methods are provided in the online version of this paper and include the following:

- KEY RESOURCES TABLE
- METHOD DETAILS
  - Single molecule definition
  - Boundary conditions
  - Berendsen barostat
  - Reactions
  - Integration of discrete and event-driven methods
  - Distribution of molecules
  - Fast algorithms for Brownian dynamics of reacting and interacting particles
  - Polydispersity
  - Simulation algorithm
  - User interface
- QUANTIFICATION AND STATISTICAL ANALYSIS

SUPPLEMENTAL INFORMATION

Supplemental information can be found online at https://doi.org/10.1016/j.crmeth.2025.101182.

Received: August 7, 2024
Revised: June 6, 2025
Accepted: August 27, 2025
Published: September 18, 2025

REFERENCES

1. Torquato, S., and Haslach Jr, H.W. (2002). Random heterogeneous materials: microstructure and macroscopic properties. Appl. Mech. Rev. *55*, B62–B63.
2. Ogarko, V., and Luding, S. (2012). A fast multilevel algorithm for contact detection of arbitrarily polydisperse objects. Comput. Phys. Commun. *183*, 931–936.
3. Helm, M.S., Dankovich, T.M., Mandad, S., Rammner, B., Jähne, S., Salimi, V., Koerbs, C., Leibrandt, R., Urlaub, H., Schikorski, T., and Rizzoli, S.O. (2021). A large-scale nanoscopy and biochemistry analysis of postsynaptic dendritic spines. Nat. Neurosci. *24*, 1151–1162.
4. Wilhelm, B.G., Mandad, S., Truckenbrodt, S., Kröhnert, K., Schäfer, C., Rammner, B., Koo, S.J., Claßen, G.A., Krauss, M., Haucke, V., et al. (2014). Composition of isolated synaptic boutons reveals the amounts of vesicle trafficking proteins. Science *344*, 1023–1028.
5. Stein, W.D. (1967). The Movement of Molecules across Cell Membranes, *6* (Academic Press).
6. S Mogre, S., Brown, A.I., and Koslover, E.F. (2020). Getting around the cell: physical transport in the intracellular world. Phys. Biol. *17*, 061003.
7. Baranovic, J. (2021). AMPA receptors in the synapse: Very little space and even less time. Neuropharmacology *196*, 108711.
8. Tanaka, H., Miyazaki, N., Matoba, K., Nogi, T., Iwasaki, K., and Takagi, J. (2012). Higher-order architecture of cell adhesion mediated by polymorphic synaptic adhesion molecules neurexin and neuroligin. Cell Rep. *2*, 101–110.
9. Kerr, R.A., Bartol, T.M., Kaminsky, B., Dittrich, M., Chang, J.C.J., Baden, S.B., Sejnowski, T.J., and Stiles, J.R. (2008). Fast Monte Carlo simulation methods for biological reaction-diffusion systems in solution and on surfaces. SIAM J. Sci. Comput. *30*, 3126–3149.
10. Stiles, J.R., Van Helden, D., Bartol, T.M., Jr., Salpeter, E.E., and Salpeter, M.M. (1996). Miniature endplate current rise times less than 100 microseconds from improved dual recordings can be modeled with passive acetylcholine diffusion from a synaptic vesicle. Proc. Natl. Acad. Sci. USA *93*, 5747–5752.
11. Stiles, J.R., and Bartol, T.M. (2001). Monte Carlo methods for simulating realistic synaptic microphysiology using MCell. In Computational Neuroscience: Realistic Modeling for Experimentalists, pp. 87–127.
12. Andrews, S.S. (2017). Smoldyn: particle-based simulation with rule-based modeling, improved molecular interaction and a library interface. Bioinformatics *33*, 710–717.
13. Hoffmann, M., Fröhner, C., and Noé, F. (2019). Readdy 2: Fast and flexible software framework for interacting-particle reaction dynamics. PLoS Comput. Biol. *15*, e1006830.
14. Gupta, S., Czech, J., Kuczewski, R., Bartol, T.M., Sejnowski, T.J., Lee, R. E., and Faeder, J.R. (2018). Spatial stochastic modeling with MCell and CellBlender. Preprint at arXiv. https://doi.org/10.48550/arXiv.1810.00499.
15. Bartol, T.M., Keller, D.X., Kinney, J.P., Bajaj, C.L., Harris, K.M., Sejnowski, T.J., and Kennedy, M.B. (2015). Computational reconstitution of spine calcium transients from individual proteins. Front. Synaptic Neurosci. *7*, 17.
16. Andrews, S.S., Addy, N.J., Brent, R., and Arkin, A.P. (2010). Detailed simulations of cell biology with Smoldyn 2.1. PLoS Comput. Biol. *6*, e1000705.
17. Andrews, S.S. (2011). Spatial and stochastic cellular modeling with the Smoldyn simulator. In Bacterial Molecular Networks: Methods and Protocols (Springer), pp. 519–542.
18. Schöneberg, J., and Noé, F. (2013). Readdy-a software for particle-based reaction-diffusion dynamics in crowded cellular environments. PLoS One *8*, e74261.
19. Fröhner, C., and Noé, F. (2018). Reversible interacting-particle reaction dynamics. J. Phys. Chem. B *122*, 11240–11250.
20. Abraham, M.J., Murtola, T., Schulz, R., Páll, S., Smith, J.C., Hess, B., and Lindahl, E. (2015). Gromacs: High performance molecular simulations through multi-level parallelism from laptops to supercomputers. SoftwareX *1–2*, 19–25.
21. Plimpton, S. (1995). Fast parallel algorithms for short-range molecular dynamics. J. Comput. Phys. *117*, 1–19.
22. Varga, M.J., Fu, Y., Loggia, S., Yogurtcu, O.N., and Johnson, M.E. (2020). Nerdss: a nonequilibrium simulator for multibody self-assembly at the cellular scale. Biophys. J. *118*, 3026–3040.
23. Ilie, I.M., Briels, W.J., and den Otter, W.K. (2015). An elementary singularity-free rotational Brownian dynamics algorithm for anisotropic particles. J. Chem. Phys. *142*, 114103.
24. García de la Torre, J., Harding, S.E., and Carrasco, B. (1999). Calculation of NMR relaxation, covolume, and scattering-related properties of bead models using the SOLPRO computer program. Eur. Biophys. J. *28*, 119–132.
25. Erban, R., and Chapman, S.J. (2009). Stochastic modelling of reaction–diffusion processes: algorithms for bimolecular reactions. Phys. Biol. *6*, 046001.
26. Trokhymchuk, A., Nezbeda, I., Jirsák, J., and Henderson, D. (2005). Hard-sphere radial distribution function again. J. Chem. Phys. *123*.
27. Tao, F.M., Song, Y., and Mason, E. (1992). Derivative of the hard-sphere radial distribution function at contact. Phys. Rev. A *46*, 8007–8008.
28. Hansen, J.P., and McDonald, I.R. (2013). Theory of Simple Liquids: With Applications to Soft Matter (Academic Press).
29. Szymczak, P., and Cieplak, M. (2011). Hydrodynamic effects in proteins. J. Phys. Condens. Matter *23*, 033102.
30. Zuk, P.J., Wajnryb, E., Mizerski, K.A., and Szymczak, P. (2014). Rotne–Prager–Yamakawa approximation for different-sized particles in application to macromolecular bead models. J. Fluid Mech. *741*, R5.
31. Ekiel-Jezewska, M., and Wajnryb, E. (2009). Precise multipole method for calculating hydrodynamic interactions between spherical particles in the stokes flow. In Theoretical Methods for Micro Scale Viscous Flows, pp. 127–172.
32. Skolnick, J. (2016). Perspective: On the importance of hydrodynamic interactions in the subcellular dynamics of macromolecules. J. Chem. Phys. *145*, 100901.
33. Geyer, T., and Winter, U. (2009). An O(N2) approximation for hydrodynamic interactions in Brownian dynamics simulations. J. Chem. Phys. *130*, 114905.
34. Fu, L., Wan, M., Zhang, S., Gao, L., and Fang, W. (2020). Polymyxin b loosens lipopolysaccharide bilayer but stiffens phospholipid bilayer. Biophys. J. *118*, 138–150.
35. Anderson, J.A., Glaser, J., and Glotzer, S.C. (2020). HOOMD-blue: A python package for high-performance molecular dynamics and hard particle Monte Carlo simulations. Comput. Mater. Sci. *173*, 109363.
36. Wu, H., Mardt, A., Pasquali, L., and Noé, F. (2018). Deep generative Markov state models. Adv. Neural Inf. Process. Syst. *31*, 3975−3984.
37. Noé, F., Tkatchenko, A., Müller, K.R., and Clementi, C. (2020). Machine learning for molecular simulation. Annu. Rev. Phys. Chem. *71*, 361–390.
38. Comer, J., and Aksimentiev, A. (2012). Predicting the DNA sequence dependence of nanopore ion current using atomic-resolution Brownian dynamics. J. Phys. Chem. C *116*, 3376–3393.

CellPress
OPEN ACCESS

39. Singharoy, A., Maffeo, C., Delgado-Magnero, K.H., Swainsbury, D.J.K., Sener, M., Kleinekathöfer, U., Vant, J.W., Nguyen, J., Hitchcock, A., Isralewitz, B., et al. (2019). Atoms to phenotypes: molecular design principles of cellular energy metabolism. Cell *179*, 1098–1111.e23.

40. Pak, A.J., and Voth, G.A. (2018). Advances in coarse-grained modeling of macromolecular complexes. Curr. Opin. Struct. Biol. *52*, 119–126.

41. Noid, W.G. (2013). Perspective: Coarse-grained models for biomolecular systems. J. Chem. Phys. *139*, 090901.

42. Ingólfsson, H.I., Lopez, C.A., Uusitalo, J.J., de Jong, D.H., Gopal, S.M., Periole, X., and Marrink, S.J. (2014). The power of coarse graining in biomolecular simulations. Wiley Interdiscip. Rev. Comput. Mol. Sci. *4*, 225–248.

43. Dignon, G.L., Zheng, W., Kim, Y.C., Best, R.B., and Mittal, J. (2018). Sequence determinants of protein phase behavior from a coarse-grained model. PLoS Comput. Biol. *14*, e1005941.

44. Espinosa, J.R., Garaizar, A., Vega, C., Frenkel, D., and Collepardo-Guevara, R. (2019). Breakdown of the law of rectilinear diameter and related surprises in the liquid-vapor coexistence in systems of patchy particles. J. Chem. Phys. *150*, 224510.

45. Tozzini, V. (2005). Coarse-grained models for proteins. Curr. Opin. Struct. Biol. *15*, 144–150.

46. Długosz, M., and Trylska, J. (2011). Diffusion in crowded biological environments: applications of Brownian dynamics. BMC Biophys. *4*, 1–9.

47. Ermak, D.L., and McCammon, J.A. (1978). Brownian dynamics with hydrodynamic interactions. J. Chem. Phys. *69*, 1352–1360.

48. Dickinson, E., Allison, S.A., and McCammon, J.A. (1985). Brownian dynamics with rotation– translation coupling. J. Chem. Soc., Faraday Trans. 2 *81*, 591–601.

49. Jones, R.B., and Pusey, P.N. (1991). Dynamics of suspended colloidal spheres. Annu. Rev. Phys. Chem. *42*, 137–169.

50. Espinosa, J.R., Vega, C., and Sanz, E. (2014). The mold integration method for the calculation of the crystal-fluid interfacial free energy from simulations. J. Chem. Phys. *141*, 134709.

51. Fang, L., Liu, J., Ju, S., Zheng, F., Dong, W., and Shen, M. (2010). Experimental and theoretical evidence of enhanced ferromagnetism in sonochemical synthesized bifeo 3 nanoparticles. Appl. Phys. Lett. *97*, 242501.

52. Shirley, P., Ashikhmin, M., and Marschner, S. (2009). Fundamentals of Computer Graphics (AK Peters/CRC Press).

53. Hu, L., Hu, G.M., Fang, Z.Q., and Zhang, Y. (2013). A new algorithm for contact detection between spherical particle and triangulated mesh boundary in discrete element method simulations. Int. J. Numer. Methods Eng. *94*, 787–804.

54. Eberly, D. (2004). 3D Game Engine Architecture: Engineering Real-Time Applications with Wild Magic (CRC Press).

55. Amanatides, J., and Woo, A. (1987). A fast voxel traversal algorithm for ray tracing. In Proceedings of Eurographics, pp. 3–10.

56. Andrews, S.S., and Bray, D. (2004). Stochastic simulation of chemical reactions with spatial resolution and single molecule detail. Phys. Biol. *1*, 137–151.

57. Ericson, C. (2004). Real-time Collision Detection (CRC Press).

58. Flegg, M.B., Chapman, S.J., and Erban, R. (2012). The two-regime method for optimizing stochastic reaction–diffusion simulations. J. R. Soc. Interface *9*, 859–868.

59. Osada, R., Funkhouser, T., Chazelle, B., and Dobkin, D. (2002). Shape distributions. ACM Trans. Graph. *21*, 807–832.

60. Muller, E.A., Ervik, A., and Mejía, A. (2020). A guide to computing interfacial properties of fluids from molecular simulations. Liv. J. Computat. Mol. Sci. *2*, 21385.

61. Espinosa, J.R., Joseph, J.A., Sanchez-Burgos, I., Garaizar, A., Frenkel, D., and Collepardo- Guevara, R. (2020). Liquid network connectivity regulates the stability and composition of biomolecular condensates with many components. Proc. Natl. Acad. Sci. USA *117*, 13238–13247.

62. Glaser, J., Zha, X., Anderson, J.A., Glotzer, S.C., and Travesset, A. (2020). Pressure in rigid body molecular dynamics. Comput. Mater. Sci. *173*, 109430.

63. Harvey, S., and Garcia de la Torre, J. (1980). Coordinate systems for modeling the hydro- dynamic resistance and diffusion coefficients of irregularly shaped rigid macromolecules. Macromolecules *13*, 960–964.

64. Erban, R., Chapman, J., and Maini, P. (2007). A practical guide to stochastic simulations of reaction-diffusion processes. Preprint at arXiv. https://doi.org/10.48550/arXiv.0704.1908.

65. Field, T., and Bradley, J. (1993). Simulation and modelling. Course Notes in Simulation and Modelling.

66. Gillespie, D.T. (1977). Exact stochastic simulation of coupled chemical reactions. J. Phys. Chem. *81*, 2340–2361.

67. Doi, M. (1976). Second quantization representation for classical many-particle system. J. Phys. A: Math. Gen. *9*, 1465–1477.

68. Polthier, K., and Schmies, M. (2006). Straightest geodesics on polyhedral surfaces. In ACM SIGGRAPH 2006 Courses (ACM), pp. 30–38.

69. Keenan, C., and Clarisse, W. (2013). Wardetzky max. Geodesics in heat: A new approach to computing distance based on heat flow. ACM Trans. Graph. *32*, 152.

70. Trettner, P., Bommes, D., and Kobbelt, L. (2021). Geodesic distance computation via virtual source propagation. Comput. Graph. Forum *40*, 247–260.

71. Michael, P., Tildesley, D., and Tildesley, D. (2017). Computer Simulation of Liquids (UK: MP Allen, Oxford Academic, DJ Tildesley).

72. Bridson, R. (2007). Fast Poisson disk sampling in arbitrary dimensions. In ACM SIGGRAPH 2007 Sketches (ACM), p. 22.

73. Corsini, M., Cignoni, P., and Scopigno, R. (2012). Efficient and flexible sampling with blue noise properties of triangular meshes. IEEE Trans. Vis. Comput. Graph. *18*, 914–924.

STAR★METHODS

KEY RESOURCES TABLE

REAGENT or RESOURCE	SOURCE	IDENTIFIER
Software and algorithms		
PyRID	This paper	https://doi.org/10.5281/zenodo.16946717 and https://github.com/MoritzB90/PyRID
Blender 2.93.9	Blender Foundation	https://www.blender.org
Cellblender 1.2	MCellTeam	https://github.com/mcellteam/cellblender
Readdy 2.0.12	Hoffmann et al.[13]	https://readdy.github.io/
MCell 4.1.0	Kerr et al.[9]	https://mcell.org/
Smoldyn 2.74	Andrews[12]	https://www.smoldyn.org/
Other		
PyRID Documentation	This paper	https://moritzb90.github.io/PyRID_doc/

METHOD DETAILS

Single molecule definition

Molecules and proteins exhibit anisotropic and multivalent interactions that cannot be accurately described by isotropic energy potentials or point-like particles.[43,44] While all-atom molecular dynamics simulations can capture protein-protein interactions with high accuracy, simulating large molecular systems over biologically relevant timescales remains computationally infeasible with current algorithms and hardware. To address this challenge, PyRID employs coarse-graining methods[45] to represent molecular structures efficiently. In this approach, rigid bead models replace strong, short-range atomic interactions with a simplified topology, enabling larger integration time steps and reducing computational costs. The beads do not necessarily correspond to individual atoms or molecules but instead approximate the overall geometry of the molecule of interest. Additionally, multivalent protein-protein interactions can be represented using surface patches on the bead model. The accuracy of such coarse-grained models depends on the choice of interaction potentials and model parameters, which must be inferred from experimental data and theoretical considerations. The motion of an isolated rigid bead molecule in solution is described by coupled Langevin equations for translational and rotational motion. However, hydrodynamic interactions between molecules are not considered due to their high computational cost.[33,46] For further details, we refer the reader to prior studies on Langevin dynamics[47–49] and the PyRID documentation.

Particle-particle interactions

PyRID can handle external potentials and pairwise, short-ranged interaction potentials.

$$F_i = \nabla_i\left(\sum_i U_{ext}(r_i) + \sum_{i\neq j} U_{pair}(r_i, r_j)\right). \quad \text{(Equation 7)}$$

$U_{ext}(r_i)$ represents the external potential acting on particle i at position r_i, while $U_{pair}(r_i, r_j)$ denotes the pairwise interaction potential between particles i and j separated by a distance r_{ij}.

PyRID has built-in functions for commonly used pairwise interaction potentials, including the weak piecewise harmonic potential,[13] harmonic repulsion potential,[13] Continuous Square-Well (CSW) potential,[50] and Pseudo Hard Sphere (PHS) potential.[51] While PyRID does not support long-range interaction potentials such as Ewald summation, users can conveniently implement custom short-range pairwise interaction potentials using Python. Similar to PyRID, ReaDDy also provides several predefined pairwise interaction potentials, including harmonic repulsion, weak interaction piecewise harmonic, Lennard-Jones, and screened electrostatics potentials. Additionally, ReaDDy supports angular constraints for particle triplets and torsion potentials for particle quadruplets, which PyRID does not currently implement. While there is some overlap in the available interaction models, PyRID focuses on a flexible and extensible implementation, allowing users to define their own interaction potentials as needed.

Compartment

Cellular processes are heavily influenced by compartmentalization, and it is desirable to confine diffusion and reactions to specific regions of interest. Various approaches can be taken to restrict the movement of particles within a confined region. One method involves placing particles along the boundary of the compartment, where they can interact with particles within the compartment via a repulsive interaction potential. Another approach would be to add external potentials/force fields that restrict the motion of particles either to the volume or the surface of a compartment. This method is used in ReaDDy.[13] However, complex geometries/compartment

shapes are more difficult to establish. A third approach represents the compartment geometry by triangulated meshes as is done in MCell.[9] This approach enables a separation of the simulation volume, e.g., into an extracellular and intracellular space. Following this approach, in PyRID a compartment is defined as a closed volume resulting from a closed surface that separates space into distinct regions. Hereby, the surface always has to form a closed volume. To enable the simulation of a section of a cell membrane (e.g., a postsynaptic spine; Figure 5G), only a part of the closed volume has to be placed within the simulation box. This allows for the confinement of diffusion and reactions within specific areas, closely mimicking biological compartmentalization. Accordingly, PyRID defines a compartment as a triangulated manifold mesh, which is efficiently stored in a shared vertex data structure[52] that includes an array of vertex positions and an array that stores triangle indices using three vertices (Figure 5A).

Volume molecules

In PyRID, two distinct methods are utilized to calculate the collision response of a molecule with a mesh. For large rigid bead molecules, collision detection involves neighbor searching and contact resolution, wherein each mesh triangle exerts a repulsive force on individual beads.[53] The accuracy and complexity of collision detection and force calculation depend on factors such as the type of geometries involved, surface complexity, and whether frictional forces must be considered. PyRID uses the "Point to Triangle" algorithm by Eberly[54] to calculate the distance between beads and triangles. For small, isotropic molecules or atoms, contact force calculation using repulsive forces requires a very small integration time step. To avoid this, PyRID utilizes ray tracing for collision detection and resolution, which works independently of integration time step (Figure 5B). This approach, similar to that used in MCell,[9] involves tracing the displacement vector of the molecule ΔR through the simulation volume and resolving collisions with the compartment boundary (mesh) via reflection.

Collision tests are performed using the 'Fast Voxel Traversal Algorithm for Ray Tracing' introduced by Amanatides et al.,[55] which has been shown to be probabilistically correct.[56] To optimally implement this method in PyRID, the continuous space is divided into cells, representing the required voxels.

Surface molecules

In PyRID, the lateral diffusion of surface molecules within the mesh surface is modeled with the aim of simulating the movement of transmembrane molecules such as receptors (Figure 5C). A method similar to that used in Smoldyn[12] is employed, wherein a molecule diffuses within the plane of a triangle until it encounters an edge. At this point, the molecule's displacement vector ΔR is advanced to the edge and then rotated into the plane of the neighboring triangle, with the rotation axis determined by the shared edge. The molecule will then move in a straight line on the mesh surface, equivalent to unfolding the triangles over the shared edge to place them in a common tangent space, advancing the position vector, and folding/rotating back. The latter view provides intuitive insight into the straight-line motion of the molecule on the mesh surface using this approach.

The method sketched above is known as "Surface Ray Marching", which we introduce briefly in the following. To effectively detect if a triangle edge has been crossed and to identify its neighboring triangle, additional data is stored alongside triangle and vertex information. Specifically, for each triangle, an array holds the vertex indices of its three edges, sorted in a counterclockwise manner, along with a separate array containing the indices of the corresponding neighboring triangles for swift reference (Figure 5A). The triangle edge intersection test is made efficient using barycentric coordinates, where the center of the molecule (R_0) and its displacement vector (ΔR) are described in terms of these coordinates. Similar to Ericson,[57] the edges are sorted counter-clockwise, starting from the triangle's origin, simplifying the edge intersection test. To determine which edge is intersected first, the smallest distance to the respective edge is checked. The process involves advancing R by $R = R_0 + t_i \Delta R$ with t_i being the distance to the intersecting edge, adjusting ΔR accordingly, and transforming R into the local coordinate frame of the neighboring triangle. Additionally, as PyRID supports anisotropic rigid bead molecules, the molecule's orientation needs updating for each crossed triangle. This is achieved by rotating the molecule using a rotation quaternion, with the quaternion propagation occurring through multiplication. The process terminates when $R_0+\Delta R$ lies within the triangle the molecule is currently located on.

In PyRID, therefore, closed volumes (compartment) are defined by user-specified closed surfaces, and molecule placement within them is determined by geometric tests such as ray tracing or bounding box intersection. These methods allow PyRID to robustly assign proteins to closed volumes and confine their dynamics accordingly. Thus, surfaces in PyRID are represented as triangulated meshes, which should be closed (enclosing a volume). Branched or non-manifold surfaces, such as dividing cells or touching soap bubbles, are currently not supported natively.

Boundary conditions

PyRID supports three types of boundary conditions: periodic, repulsive, and fixed concentration. Repulsive boundary conditions are implemented either through a repulsive interaction potential or via ray tracing, also see section "Volume molecules". When using periodic boundary condition, the minimal image convention is applied to ensure that each particle interacts only with the closest image of the other particles in the system (Figure 5H). However, it is important to note that the size of the simulation box should not be too small, as this can result in particles interacting with themselves. Since periodic and repulsive boundary conditions are common in many simulation software, we will focus on introducing the less common feature of PyRID, which is the fixed concentration boundary condition.

Fixed concentration boundary conditions

The fixed concentration boundary condition in PyRID allows for coupling the simulation box to a particle bath or molecular pool, that is outside the box (Figure 5G). This feature enables the simulation of a localized region with an imposed concentration C at the

boundary, mimicking the influence of the surrounding system without the computational demand to simulate the entire system as a particle-based or multiscale model (see Flegg et al.[58] for an example). The fixed boundary condition implies that, on the one hand, molecules can leave the simulation box forming an "outflux" while, on the other hand, new molecules are introduced into the simulation box, creating an "influx" of molecules.

Outflux. Molecules are removed from the simulation upon crossing the simulation box boundary, effectively mimicking diffusion into the external environment. Since PyRID does not directly simulate molecules outside the box, those that leave do not return or change the outside concentration C of the molecule. This approach maintains the correct steady-state concentration within the simulation region, while ensuring computational efficiency.

Influx. The fixed concentration C, outside the simulation box, is used to determine the number of molecules that will cross the simulation box boundary, without the need to simulate molecules being outside the box. Instead the fixed concentration directly influences the number of new molecules being introduced into the simulation box. For this, the expected number of hits N_{hit} between a molecule type and simulation box boundaries per time step Δt determines the number of new molecules introduced in the simulation box. N_{hit} is calculated for each molecule type based on the fixed outside concentration of the molecule C, the total boundary surface area A, and the average distance ln a diffusing molecule travels normal to a plane within Δt[9]:

$$N_{hit} = \frac{A \cdot l_n}{2C}. \quad \text{(Equation 8)}$$

The average distance l_n is calculated by

$$l_n = \sqrt{\frac{4D\Delta t}{\pi}}, \quad \text{(Equation 9)}$$

depending on the molecule's diffusion coefficient D, which is the scalar translational diffusion coefficient $D = \mathrm{Tr}(D^{tt})/3$. For surface molecules, N_{hit} is adjusted accordingly based on the boundary edge length L. Since molecular diffusion is a stochastic process, the actual number of molecules that we expect to cross each time step the boundary from the outside to be introduced in the simulation box, follows a Poisson distribution with an expected rate N_{hit}. This ensures that the introduction of new molecules into the simulation accurately reflects the probabilistic nature of diffusion.

Once the number of molecules entering the simulation box based on the fixed concentration C is determined, each new molecule needs a position to be assigned. For this, PyRID uses a probabilistic method to determine the distance between newly introduced molecules and the external boundary. The normalized distance $\tilde{x}$ of each molecule from the boundary is drawn from a probability distribution

$$P(\tilde{x}) = 1 - \exp\left(-\tilde{x}^2\right) + \sqrt{\pi} \cdot \tilde{x} \cdot \mathrm{erfc}(\tilde{x}), \quad \text{(Equation 10)}$$

leading to the distance vector $dx = \sqrt{\pi}\, l_n\, \tilde{x}\, \hat{n}$ normal to the plane by the plane's normal vector $\hat{n}$. This method ensures that molecules are placed in accordance with their expected diffusion behavior, accurately reflecting how far they would typically travel from the boundary. Once the number of molecules and their distances from the boundary are determined, they are distributed within the simulation box (see Kerr et al.[9] for more details). Since diffusion along each spatial dimension is independent, molecules are placed randomly and uniformly across the respective plane. For triangulated mesh surfaces, random points within triangles are selected, weighted by their area, ensuring an even distribution (sampling follows the method in Osada et al.[59]). If a molecule enters the simulation box close to another boundary, the distance traveled parallel to the plane must be considered to resolve collisions with the mesh, although currently, PyRID does not account for this. For small integration time steps and meshes that are further than $\sqrt{4D\Delta t}$ away from the simulation box boundary, the error introduced should, however, be negligible.

Please note that PyRID does not consider that molecules in the molecule pool outside the simulation box interact with each other. As a result, fixed concentration boundary conditions only result in identical inside and outside simulation box concentrations if no molecular interactions are simulated. Additionally, molecules within the simulation box, but being near a boundary, do not interact or react with virtual molecules across that boundary.

Berendsen barostat

In certain cases, it may be necessary to perform simulations in the NPT ensemble (constant number of particles, pressure and temperature), for instance, to release the system from stresses, compute interfacial properties of fluids,[60] or compute phase diagrams using direct coexistence methods.[44,61] PyRID supports NPT simulations using the Berendsen barostat, which scales inter-particle distances to adjust the system pressure. While this method efficiently achieves the target density, it does not strictly sample from the correct ensemble, leading to underestimated pressure fluctuations. To account for this, at each time step, molecule coordinates and the simulation box size are scaled by a factor μ dependent on the deviation of the actual pressure P from a target pressure P_0, and a coupling time constant τ_P[62]:

$$\mu = \left(1 - \Delta t/\tau_P\,(P_0 - P)\right)^{1/3}. \quad \text{(Equation 11)}$$

In isotropic systems, this applies uniformly, while for anisotropic cases, pressure is treated as a tensor, allowing independent scaling along different directions. System pressure is calculated using the virial theorem, but for rigid-body molecules, the virial must be computed using the center of diffusion rather than the center of mass.[63] This distinction arises because hydrodynamic interactions affect Brownian dynamics, meaning the effective rotation and frictional forces act through the center of diffusion rather than the geometric center of mass. For symmetric molecules, both centers coincide.

Reactions

This section introduces chemical reactions of molecules supported by PyRID (Figure 6). These reactions can include for instance post-translational modifications, ligand binding, or ATP interactions. In general, the reactions can be defined on the molecule or bead/particle level, and are categorized as uni-molecular (first order), bi-molecular (second order), or zero order reactions. Uni- and bi-molecular reactions can involve multiple reaction paths, each belonging to a different reaction type.

Unimolecular reactions

Unimolecular reactions, such as decay, fission, and conversion, can be efficiently simulated using a variation of the Gillespie Stochastic Simulation Algorithm (SSA).[64–66] This method samples the time point of the next reaction from the expected molecule lifetimes probability distribution, assuming no interfering events such as a bi-molecular reaction occur in between. The Gillespie SSA is beneficial, as it is exact and evaluates a reaction only once, instead of each time step. For a single molecule having n possible reaction paths each with a reaction rate k_i, let $k_t = \sum_i^n k_i$ be the total reaction rate. Let $\rho(\tau)d\tau$ be the probability that the next reaction occurs within $[t, t + \tau + d\tau)$, which can be split into $g(\tau)$, the probability that no reaction occurs within $[t, t + \tau)$ and probability that a reaction occurs within the time interval $d\tau$, which is given by $k_t d\tau$. Thereby,

$$\rho(\tau)d\tau = g(\tau)k_t d\tau, \quad \text{(Equation 12)}$$

where $g(\tau) = \exp(-k_t\tau)$[64]. From the above equation we find $P(\tau) = 1 - \exp(-k_t\tau)$ by integration. To sample from this distribution, we can use the inverse distribution function:

$$\tau = P^{-1}(U), \quad \text{(Equation 13)}$$

where U is uniformly distributed in $(0,1)$. With $U = P(\tau) = 1 - \exp(-k_t\tau)$, we find $P^{-1}(U) = -\log(1 - U)/k_t$. Thereby, we can draw the time point of the next reaction from:

$$\tau = \frac{1}{k_t}\ln\left[\frac{1}{U}\right]. \quad \text{(Equation 14)}$$

With the above method, we accurately sample from the distribution of expected molecule lifetimes $\rho(\tau) = k_t \exp(-k_t\tau)$. These reactions can, amongst others, describe protein degradation, ligand unbinding, disassembly of protein complexes, and changes in folded protein states. In PyRID, uni-molecular reactions can occur on either the molecule or particle level, depending on the definition of the reaction. If a conversion or decay reaction is defined on a molecule, the complete molecule is exchanged with the product or removed in the case of a decay reaction. However, if defined on a particle, only the particle is affected. PyRID offers three types of fission reactions, termed fission and production reactions defined on molecules, and release reactions defined on particles/beads (Figure S1).

Bi-molecular reactions

Due to the uncertainty of molecule encounters, bi-molecular reactions require a different approach than uni-molecular reactions. PyRID utilizes the Doi reaction scheme.[67] In this scheme, two molecules can only react if the inter-molecular distance r_{ij} is below a reaction radius R_{react}. The probability of having at least one reaction is then given by,

$$p = 1 - \exp\left(-\sum_i^n k_i \Delta t\right), \quad \text{(Equation 15)}$$

where n is the number of reaction paths.

PyRID supports various bi-molecular reactions, including fusion reactions, enzymatic reactions, and binding reactions, which can be defined on molecules and/or particles (Figure S2). For more details see the PyRID documentation.

Reaction rates in PyRID have been validated against ODE-based solutions (see Figures 1E–1G and 2), and the model accounts for steric interference via excluded volume, which slows down reactions as expected. Currently, only spherical particles are supported, and the influence of molecular orientation is not explicitly included but may be addressed in future versions.

Please note that in PyRID surface reactions between molecules on a surface are based on their Euclidean distance, which may not accurately reflect the local surface curvature. Accurately calculating geodesic distances on mesh surfaces is computationally expensive, and fast approximations such as Dijkstra's algorithm may not provide good approximations for points close to the source. In addition, as reaction radii are typically small compared to the mesh size and surface curvature, deviations in calculations are expected to be small in most cases. Nonetheless, recent progress has been made in this area[68–70] and could be implemented into PyRID in future versions.

Integration of discrete and event-driven methods

In PyRID, time advances in discrete steps while space is treated continuously, to ensure a balance between accuracy and computational efficiency. Features employing event-driven methods, like the Gillespie method for unimolecular reactions, are scheduled such that they take place at specific time steps. Similarly, features based on fixed time steps, like the Doi reaction scheme for bimolecular reactions, are also scheduled in PyRID for happening at specific time steps. By combining discrete time steps with continuous spatial representation and integrating both event-driven and fixed time-step methods, PyRID is based on a hybrid approach that optimizes the numerical simulation process in a flexible and efficient framework for simulating complex molecular systems.

Distribution of molecules

Volume molecules

When dealing with mesh compartments and accounting for molecule excluded volume, distributing molecules in the simulation volume becomes a challenging task. A standard approach involves loosely distributing molecules and shrinking the volume until reaching a target density. This approach could be transferred to a system with mesh compartments. However, here, we might also care about the exact dimensions of the compartment so that the synchronous convergence of the desired dimensions and the molecular density becomes an additional challenge. Alternatively, the Metropolis Monte Carlo method can be used,[71] but it is time-consuming. Instead, PyRID employs the Poisson-Disc sampling algorithm,[72] which is computationally efficient and straightforward to implement but has a density limit of 30% and is only suitable for spherical molecules. For highly aspherical molecules, Monte-Carlo sampling is used, and overlaps are resolved via a soft repulsive potential. The Berendsen barostat at high pressure can also drive the system to a high-density state if no mesh compartments are used.

The Poisson-Disc sampling algorithm consists of initializing a grid, creating a sample point, and inserting it into a list of active elements. Random points around the annulus of the active sample points are created, and new sample points are accepted and inserted into the grid and active list if no other sample points exist within the radius. If no new sample point is found after k trials, the active sample point is removed from the active list. PyRID extends this algorithm to account for polydisperse particle distributions (see Figure 5I).

Surface molecules

We use an algorithm introduced by Corsini et al.[73] to distribute molecules on the surface of a mesh compartment. The algorithm involves generating a sample pool, dividing space into cells, randomly selecting cells and samples, checking distances between occupied and unoccupied samples, and updating the number of samples for each cell. The process is repeated until the desired number of molecules is reached or a maximum number of trials is reached. PyRID also allows the user to assign surface regions for molecule distribution. Example is shown in Figures 5J and 5K.

Fast algorithms for Brownian dynamics of reacting and interacting particles

The simulation performance in PyRID is optimized using Numba's jit compilation. Additionally, PyRID employs a data-oriented design and dynamic array data structures to efficiently track molecules and their reactions. PyRID requires a data structure capable of efficiently handling the constant changes in the number of particles and molecules within the system due to reactions and events. Similarly, the molecular reactions occurring at each time step must be listed and evaluated efficiently. To achieve this, PyRID utilizes two variants of dynamic array data structures, namely the tightly packed dynamic array and the dynamic array with holes, both well-suited for these tasks.

The tightly packed dynamic array (dense array)

A tightly packed dynamic array is a variant of dynamic arrays used in PyRID that enables efficient deletion of elements via a pop and swap mechanism. Unlike standard numpy arrays, lists, or C++ vectors, the tightly packed dynamic array does not create a new array every time an element is deleted or appended, which significantly reduces computational cost. This is achieved by increasing the array size by a multiplicative factor and keeping track of the length and capacity of the array. However, this method requires keeping track of the location of elements in the array as they move around during deletion or swapping, which is accomplished by maintaining a second array (see Figure S3).

The dynamic array with holes

The dynamic array with holes method (Figure S3) is used to store molecules and particles, where holes are created to enable efficient deletion, tracked via a free linked list. This approach preserves the original indices and allows for fast element access. However, if the array is sparse, it may not be cache-friendly and iterating over elements becomes complex. To address this, a tightly packed array is added to store occupied slot indices for efficient iteration. The strength of this method lies in its ability to preserve original indices while enabling fast element access and efficient deletion.

Dynamic arrays used for reaction handling

The data structure required to organize reactions is more complex than a simple dynamic array as used for rigid body molecules and particles. To efficiently add, delete single and all reactions of a particle, and return a random reaction from the list, a combination of dynamic arrays and a hash table is used. A doubly linked list is embedded into a dynamic array with holes to connect all reactions of a particle, and its head is saved in a hash table. A 4-dimensional next and previous pointer is required to keep track of the educts in a reaction. A separate dynamic array is used to keep track of all remaining reactions in a tightly packed format for easy random selection. The proposed data structure avoids introducing biases and efficiently performs the required operations.

Polydispersity

In molecular dynamics simulations, the polydispersity of particle radii can pose a problem, particularly when using minimal coarse-graining approaches with low granularity. This can lead to classical linked cell list algorithms becoming inefficient. The most computationally expensive task in such simulations is typically the calculation of pairwise interaction forces, which requires determining the distance between particles. Naively iterating over all particle pairs results in a quadratic increase in computation time with the number of particles in the system. The linked cell list approach provides a straightforward solution to this problem.[71] By dividing the simulation box into cells of appropriate size, the computational complexity can be reduced to a more linear increase with N. The number of cells must be chosen such that the side length of the cells in each dimension is larger than the maximum cutoff radius for pairwise molecular interactions. The resulting computation time is of the order of N instead of quadratic N^2, assuming a mostly homogeneous distribution of molecules.

However, this approach does not efficiently handle polydisperse particle size distributions, which can result in inefficient calculations when using minimal coarse-graining approaches with low granularity. To address this issue, a hierarchical grid approach has been introduced by Ogarko and Luding.[2] In this approach, which is used in PyRID, each particle is assigned to a cell grid based on its cutoff radius, resulting in different levels or hierarchies, each with a different cell size. Although this approach requires more memory, it significantly reduces the number of distance calculations required for larger particles and takes advantage of Newton's third law. The algorithm involves assigning particles to cells on their respective level, performing distance checks for nearest neighbor cells on the same level, and then doing a cross-level search using distance checks only on lower hierarchy levels. However, this approach may require iterating over many empty cells during the cross-level search.

While the hierarchical grid method adds some algorithmic complexity, it significantly improves efficiency for highly polydisperse systems—by about a factor of six as shown in Figure 4. Particles are assigned to different grid levels based on their cutoff radius, creating multiple hierarchies with varying cell sizes. This significantly reduces the number of required distance calculations for larger particles. The algorithm performs nearest-neighbor checks within each hierarchical level using a linked-cell approach, followed by cross-level checks only against smaller particle levels. Despite some overhead from searching empty cells, this method maintains nearly the same performance for polydisperse systems as for monodisperse ones. For more details, see Ogarko and Luding.[2]

Simulation algorithm

The simulation loop of PyRID begins by updating the position of all particles, followed by determining the particle pair distances and calculating the forces, which are then added to the reactions list. Reactions are performed next, allowing new particles to enter the system through fusion or fission reactions. To save computation time, the inter-particle distances and forces are not updated, except for binding reactions where the force is calculated at the time of reaction execution. In the next iteration, the reaction products diffuse freely without experiencing any forces yet, and the trajectory of particles that were already in the system, remains unchanged due to the presence of new molecules. The placement of product molecules does not resolve any collisions with nearby molecules, introducing a small error. Fission reactions have a similar error regardless of whether forces are updated or not. For fusion reactions, the error is slightly different as the educts leave behind an empty space that will be filled with new molecules. To avoid issues with a dense system, the integration time step should be decreased.

An alternative approach would be to calculate all forces and reactions at the beginning of each iteration before updating molecule positions. However, this method can introduce bias, especially for bimolecular reactions. PyRID's approach avoids bias when interactions are not considered. ReaDDy updates the neighboring list for molecules twice per iteration, which could double computation time if not optimized. PyRID evaluates forces and reactions in one loop over all particle pairs in the neighboring list, with pair distances calculated only once. Future work could optimize the update of pair distances after reactions are executed.

User interface

Model setup, including defining molecular interactions, reaction networks, and simulation parameters, is performed through Python scripts. Hereby, to enhance accessibility and ease of use, PyRID has been developed with a flexible interface that supports simulations via Jupyter notebooks, standalone Python scripts, or integration into larger workflows. The Github repository documentation includes several example cases that illustrate the complete workflow from system setup to analysis.

For visualization, we have developed a Blender add-on for PyRID (Figure 7), which is used exclusively for rendering simulation results. Additionally, PyRID supports reaction network visualization using graph representations (Figure S4). This feature is built upon the pyvis library, enabling interactive exploration of reaction pathways within the simulation.

QUANTIFICATION AND STATISTICAL ANALYSIS

Statistical details of simulation results and related parameter values, number, etc. can be found in the figure legends and related results section.

Cell Reports Methods, Volume *5*

Supplemental information

The Brownian dynamics simulator PyRID for reacting and interacting particles written in Python

Moritz Becker, Nahid Safari, and Christian Tetzlaff

Supplementary Document S1

The Brownian dynamics simulator PyRID for reacting and interacting particles written in Python

Moritz Becker, Nahid Safari, and Christian Tetzlaff

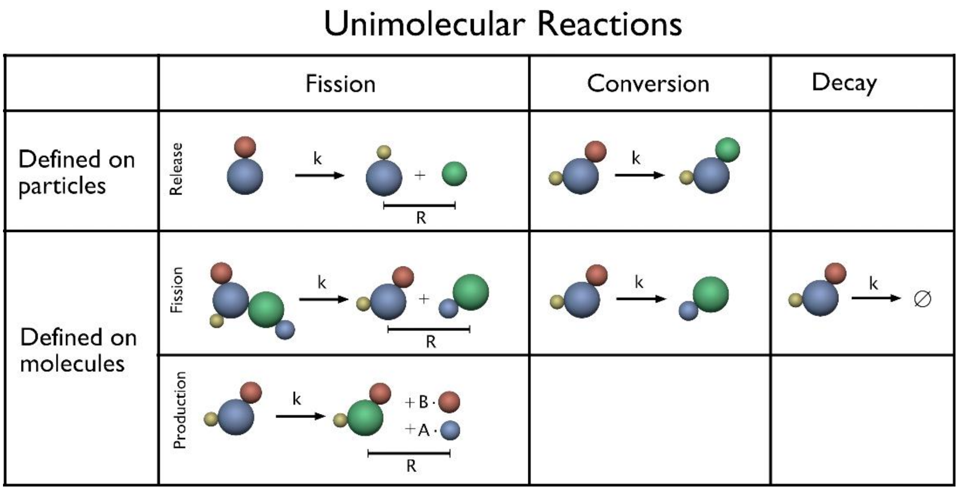

Figure S1. Schematic of unimolecular reactions. Related to Figure 6.
Unimolecular reactions can be either defined on a particle or on a molecule type. Release reactions are a sub-category of the fission reaction. Here, a molecule (rigid bead model) is released from another molecular particle/bead. Release reactions can be used, for example, to model the release of a ligand from a specific molecule's binding site. The production reaction enables a fission reaction with more than two products. It can be used, for example, to model the influx of ions into a compartment via an ion channel.

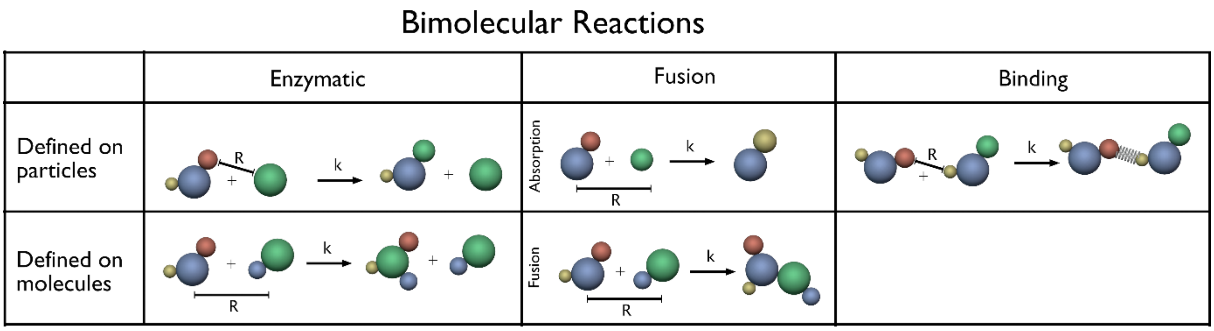

Figure S2. Schematic of bimolecular reactions. Related to Figure 6.
Bimolecular reactions can be either defined on a particle or on a molecule type. The absorption reaction is a sub-category of the fusion reaction. It has been introduced as the inverse to the release reaction and can be used to model, e.g., ligand binding. Here, a molecule is absorbed by the bead/particle of another molecule and thus removed from the simulation. Binding reactions introduce an energy potential between two particles/beads. Short-range interactions can be used, for example, to model patchy particles.

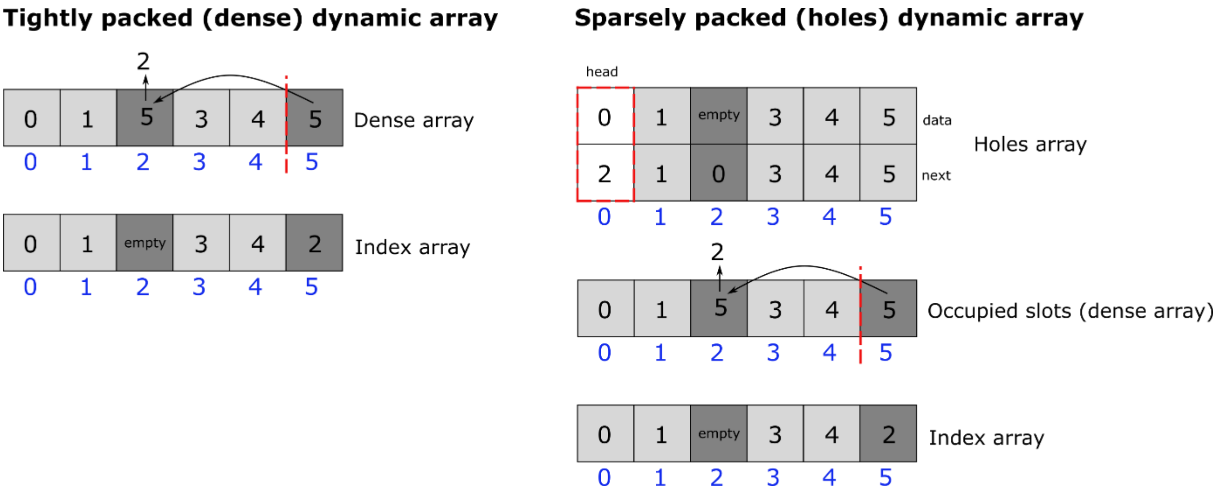

Figure S3. Dynamic arrays. Related to STAR Methods.
(left) Tightly packed dynamic array. (right) Sparsely packed dynamic arrays.

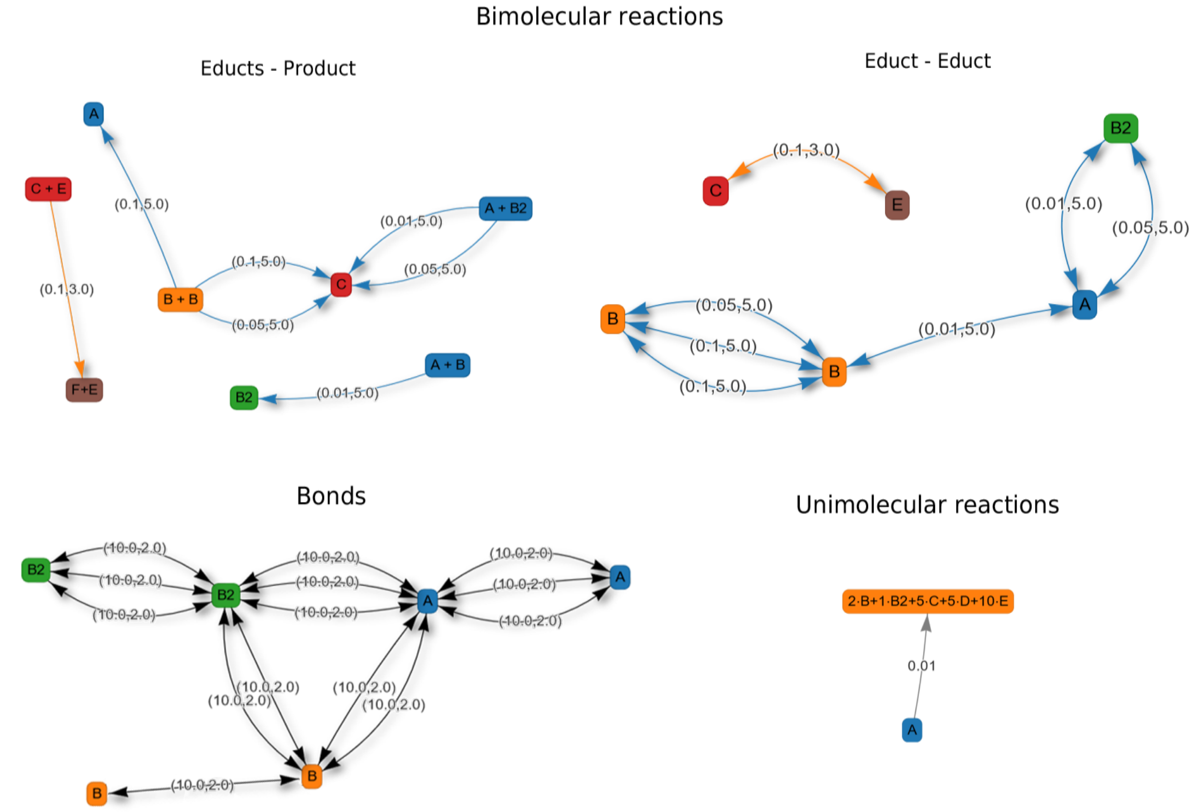

Figure S4. Examples of different reaction graphs. Related to Figure 7.
The ”Educts - Product” graph shows the reactants and their respective products as well as the various possible reaction paths. The reaction rate and the reaction radius are shown in round brackets. The ”Educt - Educt” graph only depicts the relationship between the different educts. The ”Bonds” graph shows the binding reactions between particle pairs.
